# Heterologous biosynthesis and manipulation of crocetin in *Saccharomyces cerevisiae*

**DOI:** 10.1186/s12934-017-0665-1

**Published:** 2017-03-29

**Authors:** Fenghua Chai, Ying Wang, Xueang Mei, Mingdong Yao, Yan Chen, Hong Liu, Wenhai Xiao, Yingjin Yuan

**Affiliations:** 1 Key Laboratory of Systems Bioengineering (Ministry of Education), Tianjin University, 92, Weijin Road, Nankai District, Tianjin, 300072 People’s Republic of China; 2 SynBio Research Platform, Collaborative Innovation Center of Chemical Science and Engineering (Tianjin), School of Chemical Engineering and Technology, Tianjin University, Tianjin, 300072 People’s Republic of China

**Keywords:** Metabolic engineering, Crocetin, *Saccharomyces cerevisiae*, Synthetic biology, Enzyme sources

## Abstract

**Background:**

Due to excellent performance in antitumor, antioxidation, antihypertension, antiatherosclerotic and antidepressant activities, crocetin, naturally exists in *Crocus sativus* L., has great potential applications in medical and food fields. Microbial production of crocetin has received increasing concern in recent years. However, only a patent from EVOVA Inc. and a report from Lou et al. have illustrated the feasibility of microbial biosynthesis of crocetin, but there was no specific titer data reported so far. *Saccharomyces cerevisiae* is generally regarded as food safety and productive host, and manipulation of key enzymes is critical to balance metabolic flux, consequently improve output. Therefore, to promote crocetin production in *S. cerevisiae,* all the key enzymes, such as CrtZ, CCD and ALD should be engineered combinatorially.

**Results:**

By introduction of heterologous CrtZ and CCD in existing β-carotene producing strain, crocetin biosynthesis was achieved successfully in *S. cerevisiae*. Compared to culturing at 30 °C, the crocetin production was improved to 223 μg/L at 20 °C. Moreover, an optimal CrtZ/CCD combination and a titer of 351 μg/L crocetin were obtained by combinatorial screening of CrtZs from nine species and four CCDs from *Crocus*. Then through screening of heterologous ALDs from *Bixa orellana* (Bix_ALD) and *Synechocystis* sp. PCC6803 (Syn_ALD) as well as endogenous ALD6, the crocetin titer was further enhanced by 1.8-folds after incorporating Syn_ALD. Finally a highest reported titer of 1219 μg/L at shake flask level was achieved by overexpression of CCD2 and Syn_ALD. Eventually, through fed-batch fermentation, the production of crocetin in 5-L bioreactor reached to 6278 μg/L, which is the highest crocetin titer reported in eukaryotic cell.

**Conclusions:**

*Saccharomyces cerevisiae* was engineered to achieve crocetin production in this study. Through combinatorial manipulation of three key enzymes CrtZ, CCD and ALD in terms of screening enzymes sources and regulating protein expression level (reaction temperature and copy number), crocetin titer was stepwise improved by 129.4-fold (from 9.42 to 1219 μg/L) as compared to the starting strain. The highest crocetin titer (6278 μg/L) reported in microbes was achieved in 5-L bioreactors. This study provides a good insight into key enzyme manipulation involved in serial reactions for microbial overproduction of desired compounds with complex structure.

**Electronic supplementary material:**

The online version of this article (doi:10.1186/s12934-017-0665-1) contains supplementary material, which is available to authorized users.

## Background

**Fig. 1:**
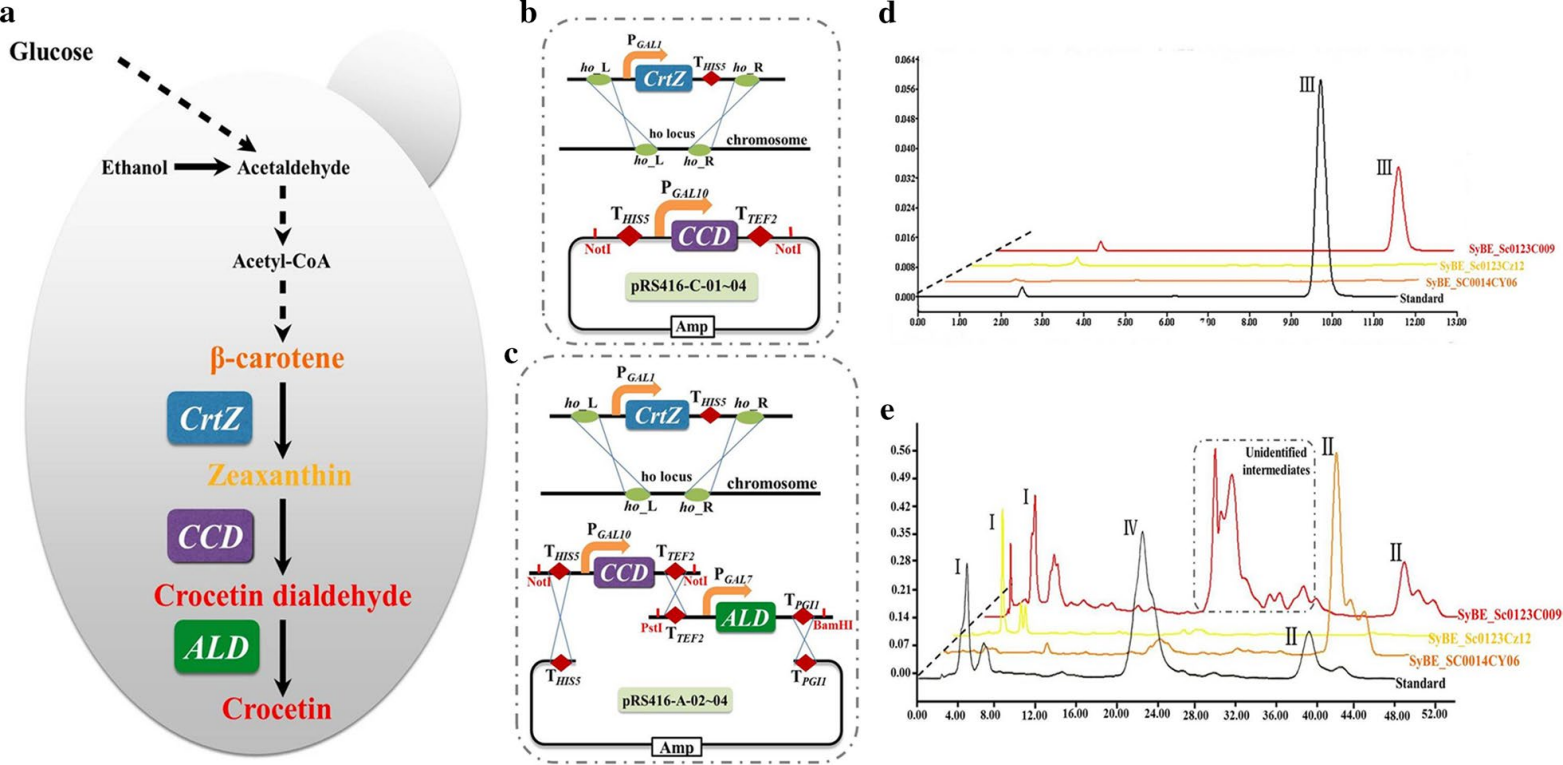
Crocetin biosynthesis pathway construction in *S. cerevisiae*. **a** The paradigm of crocetin biosynthetic pathway in *S. cerevisiae*. The synthetic pathway to crocetin from β-carotene consists of three enzymes: CrtZ, β-carotene hydroxylase; CCD, carotenoid cleavage dioxygenase and ALD, aldehyde dehydrogenase. **b**, **c** Schematic representation of the engineering strategies for CrtZ, CCD and ALD expression cassette. CrtZ expression cassette was integrated into the *ho* locus of the chromosome, while CCD or CCD/ALD was carried by centromeric plasmid pRS416. *ho*_L, *ho* locus left homologous arm; *ho*_R, *ho* locus right homologous arm. **d**, **e** The HPLC profile of the parent strain SyBE_Sc0014CY06 (*orange*), zeaxanthin producing strain SyBE_Sc0123Cz12 (*yellow*), crocetin producing strain SyBE_Sc0123C009 (*red*), and standard (*black*). The signals for zeaxanthin (I), β-carotene (II) and lycopene (IV) were detected at 450 nm, while crocetin (III) was at 430 nm. The retention time of the unidentified intermediates which were boxed was close to that of lycopene

Crocetin, a kind of carotenoid existing in *Crocus sativus* L. [1], has great potential medical applications due to various pharmacological activities, such as antitumor [2, 3], antioxidation [4], antihypertension [5], antiatherosclerotic [6] and antidepressant [7]. Additionally, crocetin can be also used as edible pigment. Currently, since crocetin manufacture mainly relied on extraction and purification from *Crocus* stigmas, deficient resource and low extraction rate restricted the large-scale application for commercialization. De novo synthesis of crocetin from simple carbon (glucose etc.) in engineered heterologous hosts would be an important complement to traditional sources. For crocetin biosynthesis, the conversion of β-carotene to crocetin required three steps catalyzed by β-carotene hydroxylase (CrtZ), carotenoid cleavage dioxygenase (CCD) and aldehyde dehydrogenase (ALD), respectively (Fig. 1a) [8]. It is speculated that balancing metabolic flux mediated by the above three enzymes is a big challenge for high output. To date, only a patent from EVOVA Inc. [9] and a report from Lou et al. [10] have just illustrated the feasibility of heterologous biosynthesis of crocetin, and there was no specific titer data reported yet. For promoting crocetin production, combinatorial manipulation of the CrtZ, CCD and ALD would be a promising solution to overcome this challenge.

Screening enzymes sources and regulating protein expression level have been proved to be efficient strategies for manipulating the key enzymes for balancing metabolic flux, consequently improving production [11–13]. Cao et al. [14] once improved odd-chain fatty alcohols production in *Escherichia coli* through balancing the expression level of TesA, αDOX, AHRs and the genes involved in fatty acids metabolism pathway. Meanwhile, through combinatorially screening the carotenogenic enzymes (CrtE, CrtB and CrtI) from diverse organisms and fine-tuning the expression level of CrtI, an optimal enzymes combination with the highest lycopene yield was obtained in *Saccharomyces cerevisiae* [15]. In crocetin biosynthesis fields, CrtZ, CCD and ALD have been characterized separately in the last decades. Li et al. [16] once achieved zeaxanthin titer as 43.46 mg/L in a recombinant *E. coli* strain by integrating *Pantoea ananatis* CrtZ into a β-carotene producing strain. Meanwhile, *Crocus* ZCD was firstly annotated as 7, 8 (7′, 8′)-zeaxanthin cleavage dioxygenase in 2003 [17]. However, Frusciante et al. [18] demonstrated this enzyme could not achieve crocetin synthesis in *E. coli* and corn. Another two *Crocus* CCDs, CCD2 [18] and ZCD1 [10], have been proved to cleavage of zeaxanthin at the 7, 8- and 7′, 8′-positions for forming crocetin dialdehyde in *E. coli* and *Chlorella vulgaris*, respectively. Moreover, even though EVOVA Inc. [9] and Lou et al. [10] realized crocetin synthesis by using endogenous ALD in yeast and algae, respectively, there was no titer data uncovered yet. It could guess that the complexity of fine-tuning *CrtZ, CCD and ALD* was the main obstacle. Therefore, it is urgent to explore *CrtZ*, *CCD* and *ALD* systematically for crocetin higher production.

*Saccharomyces cerevisiae* has been reported as a safe (Generally Recognized as Safe, GRAS) and robust host cell to produce heterologous carotenoids, including lycopene [19], β-carotene [20] and astaxanthin [21]. Thus, in our study, crocetin was successfully synthesized in *S. cerevisiae* through incorporating heterologous CrtZ and CCD in an existing β-carotene producing strain SyBE_Sc0014CY06 (with β-carotene titer of 220 mg/L) (Table 1). A higher crocetin titer was achieved by adjusting the culture temperature from 30 to 20 °C. The production of crocetin was further enhanced by 2.8-fold via screening of CrtZ/CCD combination and ALD sources. Moreover, the crocetin titer was reached to 1219 μg/L by increasing the copy numbers of *ccd* and *ald*. Finally, the highest reported crocetin titer as 6278 μg/L was archived in 5-L bioreactors. This study sets a good example of fine-tuning multiple enzymes systematically for heterologous biosynthesis of desired pharmaceuticals and chemicals.

**Table 1:** *S. cerevisiae* strains and plasmids used in this study

	Description	Source
Strain		
CEN.PK2-1C	*MATa*, *ura3*-*52*, *trp1*-*289*, *leu2*-*3,112*, *his3Δ1*, *MAL2*-*8C*, *SUC2*	EUROSCARF
SyBE_Sc0014CY06	CEN.PK2-1C, *Δgal1 Δgal7 Δgal10::HIS3, Δypl062w::KanMX, trp1::TRP1_*T_*CYC1*_-Bt*CrtI*-P_*GAL10*_-P_*GAL1*_-Pa*CrtB*-T_*PGK1*_, *leu2::LEU2_*T_*CYC1*_-Bt*CrtI*-P_*GAL7*_-T_*ACT1*_-*tHMG1*-P_*GAL10*_-P_*GAL1*_-Tm*CrtE*-T_*GPM1*_ *, Δymrwdelta15::*P_*UAS*-*GAL1*_-Pa*CrtY*-T_*ADH1*_ *, Δynrcdelta9::* P_*UAS*-*GAL1*_-Pa*CrtY*-T_*ADH1*_	This lab
SyBE_Sc0123Z001	SyBE_SC0014CY06, *Δho::*P_*GAL1*_-Aa*_CrtZ*-T_*HIS5*_-*URA3*	This study
SyBE_Sc0123Z002	SyBE_SC0014CY06, *Δho::*P_*GAL1*_-As*_CrtZ*-T_*HIS5*_-*URA3*	This study
SyBE_Sc0123Z003	SyBE_SC0014CY06, *Δho::*P_*GAL1*_-Eu*_CrtZ*-T_*HIS5*_-*URA3*	This study
SyBE_Sc0123Z004	SyBE_SC0014CY06, *Δho::*P_*GAL1*_-Pa*_CrtZ*-T_*HIS5*_-*URA3*	This study
SyBE_Sc0123Z005	SyBE_SC0014CY06, *Δho::*P_*GAL1*_-Ps*_CrtZ*-T_*HIS5*_ -*URA3*	This study
SyBE_Sc0123Z006	SyBE_SC0014CY06, *Δho::*P_*GAL1*_-Ss*_CrtZ*-T_*HIS5*_-*URA3*	This study
SyBE_Sc0123Z007	SyBE_SC0014CY06, *Δho::*P_*GAL1*_-B.SD*_CrtZ*-T_*HIS5*_-*URA3*	This study
SyBE_Sc0123Z008	SyBE_SC0014CY06, *Δho::*P_*GAL1*_-B.DC*_CrtZ*-T_*HIS5*_-*URA3*	This study
SyBE_Sc0123Z009	SyBE_SC0014CY06, *Δho::*P_*GAL1*_-Hp*_CrtZ*-T_*HIS5*_-*URA3*	This study
SyBE_Sc0123Cz10	SyBE_SC0014CY06, *Δho::*P_*GAL1*_-Aa*_CrtZ*-T_*HIS5*_	This study
SyBE_Sc0123Cz11	SyBE_SC0014CY06, *Δho::*P_*GAL1*_-As*_CrtZ*-T_*HIS5*_	This study
SyBE_Sc0123Cz12	SyBE_SC0014CY06, *Δho::*P_*GAL1*_-Eu*_CrtZ*-T_*HIS5*_	This study
SyBE_Sc0123Cz13	SyBE_SC0014CY06, *Δho::*P_*GAL1*_-Pa*_CrtZ*-T_*HIS5*_	This study
SyBE_Sc0123Cz14	SyBE_SC0014CY06, *Δho::*P_*GAL1*_-Ps*_CrtZ*-T_*HIS5*_	This study
SyBE_Sc0123Cz15	SyBE_SC0014CY06, *Δho::*P_*GAL1*_-Ss*_CrtZ*-T_*HIS5*_	This study
SyBE_Sc0123Cz16	SyBE_SC0014CY06, *Δho::*P_*GAL1*_-B.SD*_CrtZ*-T_*HIS5*_	This study
SyBE_Sc0123Cz17	SyBE_SC0014CY06, *Δho::*P_*GAL1*_-B.DC*_CrtZ*-T_*HIS5*_	This study
SyBE_Sc0123Cz18	SyBE_SC0014CY06, *Δho::*P_*GAL1*_-Hp*_CrtZ*-T_*HIS5*_	This study
SyBE_Sc0123C001	SyBE_Sc0123Cz10 with pRS416-C-01 (pRS416-T_*HIS5*_-P_*GAL10*_-*CCD2*-T_*TEF2*_)	This study
SyBE_Sc0123C002	SyBE_Sc0123Cz10 with pRS416-C-02 (pRS416-T_*HIS5*_-P_*GAL10*_-*CCD3*-T_*TEF2*_)	This study
SyBE_Sc0123C003	SyBE_Sc0123Cz10 with pRS416-C-03 (pRS416-T_*HIS5*_-P_*GAL10*_-*ZCD*-T_*TEF2*_)	This study
SyBE_Sc0123C004	SyBE_Sc0123Cz10 with pRS416-C-04 (pRS416-T_*HIS5*_-P_*GAL10*_-*ZCD1*-T_*TEF2*_)	This study
SyBE_Sc0123C005	SyBE_Sc0123Cz11 with pRS416-C-01 (pRS416-T_*HIS5*_-P_*GAL10*_-*CCD2*-T_*TEF2*_)	This study
SyBE_Sc0123C006	SyBE_Sc0123Cz11 with pRS416-C-02 (pRS416-T_*HIS5*_-P_*GAL10*_-*CCD3*-T_*TEF2*_)	This study
SyBE_Sc0123C007	SyBE_Sc0123Cz11 with pRS416-C-03 (pRS416-T_*HIS5*_-P_*GAL10*_-*ZCD*-T_*TEF2*_)	This study
SyBE_Sc0123C008	SyBE_Sc0123Cz11 with pRS416-C-04 (pRS416-T_*HIS5*_-P_*GAL10*_-*ZCD1*-T_*TEF2*_)	This study
SyBE_Sc0123C009	SyBE_Sc0123Cz12 with pRS416-C-01 (pRS416-T_*HIS5*_-P_*GAL10*_-*CCD2*-T_*TEF2*_)	This study
SyBE_Sc0123C010	SyBE_Sc0123Cz12 with pRS416-C-02 (pRS416-T_*HIS5*_-P_*GAL10*_-*CCD3*-T_*TEF2*_)	This study
SyBE_Sc0123C011	SyBE_Sc0123Cz12 with pRS416-C-03 (pRS416-T_*HIS5*_-P_*GAL10*_-*ZCD*-T_*TEF2*_)	This study
SyBE_Sc0123C012	SyBE_Sc0123Cz12 with pRS416-C-04 (pRS416-T_*HIS5*_-P_*GAL10*_-*ZCD1*-T_*TEF2*_)	This study
SyBE_Sc0123C013	SyBE_Sc0123Cz13 with pRS416-C-01 (pRS416-T_*HIS5*_-P_*GAL10*_-*CCD2*-T_*TEF2*_)	This study
SyBE_Sc0123C014	SyBE_Sc0123Cz13 with pRS416-C-02 (pRS416-T_*HIS5*_-P_*GAL10*_-*CCD3*-T_*TEF2*_)	This study
SyBE_Sc0123C015	SyBE_Sc0123Cz13 with pRS416-C-03 (pRS416-T_*HIS5*_-P_*GAL10*_-*ZCD*-T_*TEF2*_)	This study
SyBE_Sc0123C016	SyBE_Sc0123Cz13 with pRS416-C-04 (pRS416-T_*HIS5*_-P_*GAL10*_-*ZCD1*-T_*TEF2*_)	This study
SyBE_Sc0123C017	SyBE_Sc0123Cz14 with pRS416-C-01 (pRS416-T_*HIS5*_-P_*GAL10*_-*CCD2*-T_*TEF2*_)	This study
SyBE_Sc0123C018	SyBE_Sc0123Cz14 with pRS416-C-02 (pRS416-T_*HIS5*_-P_*GAL10*_-*CCD3*-T_*TEF2*_)	This study
SyBE_Sc0123C019	SyBE_Sc0123Cz14 with pRS416-C-03 (pRS416-T_*HIS5*_-P_*GAL10*_-*ZCD*-T_*TEF2*_)	This study
SyBE_Sc0123C020	SyBE_Sc0123Cz14 with pRS416-C-04(pRS416-*T*_*HIS5*_-*P*_*GAL10*_-*ZCD1*-*T*_*TEF2*_)	This study
SyBE_Sc0123C021	SyBE_Sc0123Cz15 with pRS416-C-01 (pRS416-T_*HIS5*_-P_*GAL10*_-*CCD2*-T_*TEF2*_)	This study
SyBE_Sc0123C022	SyBE_Sc0123Cz15 with pRS416-C-02 (pRS416-T_*HIS5*_-P_*GAL10*_-*CCD3*-T_*TEF2*_)	This study
SyBE_Sc0123C023	SyBE_Sc0123Cz15 with pRS416-C-03 (pRS416-T_*HIS5*_-P_*GAL10*_-*ZCD*-T_*TEF2*_)	This study
SyBE_Sc0123C024	SyBE_Sc0123Cz15 with pRS416-C-04 (pRS416-T_*HIS5*_-P_*GAL10*_-*ZCD1*-T_*TEF2*_)	This study
SyBE_Sc0123C025	SyBE_Sc0123Cz16 with pRS416-C-01 (pRS416-T_*HIS5*_-P_*GAL10*_-*CCD2*-T_*TEF2*_)	This study
SyBE_Sc0123C026	SyBE_Sc0123Cz16 with pRS416-C-02 (pRS416-T_*HIS5*_-P_*GAL10*_-*CCD3*-T_*TEF2*_)	This study
SyBE_Sc0123C027	SyBE_Sc0123Cz16 with pRS416-C-03 (pRS416-T_*HIS5*_-P_*GAL10*_-*ZCD*-T_*TEF2*_)	This study
SyBE_Sc0123C028	SyBE_Sc0123Cz16 with pRS416-C-04 (pRS416-T_*HIS5*_-P_*GAL10*_-*ZCD1*-T_*TEF2*_)	This study
SyBE_Sc0123C029	SyBE_Sc0123Cz17 with pRS416-C-01 (pRS416-T_*HIS5*_-P_*GAL10*_-*CCD2*-T_*TEF2*_)	This study
SyBE_Sc0123C030	SyBE_Sc0123Cz17 with pRS416-C-02 (pRS416-T_*HIS5*_-P_*GAL10*_-*CCD3*-T_*TEF2*_)	This study
SyBE_Sc0123C031	SyBE_Sc0123Cz17 with pRS416-C-03 (pRS416-T_*HIS5*_-P_*GAL10*_-*ZCD*-T_*TEF2*_)	This study
SyBE_Sc0123C032	SyBE_Sc0123Cz17 with pRS416-C-04 (pRS416-T_*HIS5*_-P_*GAL10*_-*ZCD1*-T_*TEF2*_)	This study
SyBE_Sc0123C033	SyBE_Sc0123Cz18 with pRS416-C-01 (pRS416-T_*HIS5*_-P_*GAL10*_-*CCD2*-T_*TEF2*_)	This study
SyBE_Sc0123C034	SyBE_Sc0123Cz18 with pRS416-C-02 (pRS416-T_*HIS5*_-P_*GAL10*_-*CCD3*-T_*TEF2*_)	This study
SyBE_Sc0123C035	SyBE_Sc0123Cz18 with pRS416-C-03 (pRS416-T_*HIS5*_-P_*GAL10*_-*ZCD*-T_*TEF2*_)	This study
SyBE_Sc0123C036	SyBE_Sc0123Cz18 with pRS416-C-04 (pRS416-T_*HIS5*_-P_*GAL10*_-*ZCD1*-T_*TEF2*_)	This study
SyBE_Sc0123C048	SyBE_Sc0123Cz14 with pRS416-A-02 (pRS416-T_*HIS5*_-P_*GAL10*_-*CCD2*-T_*TEF2*_-P_*GAL7*_-*ALD6*-T_*PGI1*_ *)*	This study
SyBE_Sc0123C049	SyBE_Sc0123Cz14 with pRS416-A-03 (pRS416-T_*HIS5*_-P_*GAL10*_-*CCD2*-T_*TEF2*_-P_*GAL7*_- Bix*_ALD*-T_*PGI1*_ *)*	This study
SyBE_Sc0123C050	SyBE_Sc0123Cz14 with pRS416-A-04 (pRS416-T_*HIS5*_-P_*GAL10*_-*CCD2*-T_*TEF2*_-P_*GAL7*_- Syn*_ALD*-T_*PGI1*_ *)*	This study
SyBE_Sc0123C053	SyBE_Sc0123Cz14 with pRS426-A-02 (pRS426-T_*HIS5*_-P_*GAL10*_-*CCD2*-T_*TEF2*_-P_*GAL7*_- Syn*_ALD*-T_*PGI1*_ *)*	This study
Plasmid		
pJET1.2	Blunt Cloning vector, resistant to ampicillin	Thermo scientific
pUC57-Simple	Blunt Cloning vector, resistant to ampicillin	GenScript
pRS416	Single copy plasmid in *S.cerevisiae* with *URA3* and Amp^r^ marker	This Lab
pRS426	Multiple copy plasmid in *S.cerevisiae* with *URA3* and Amp^r^ marker	This Lab
pRS425 K	Multiple copy plasmid in *S.cerevisiae* with *LEU2* and KanMX marker	This Lab
pUC57-Simple-01	*CrtZ* from *Agrobacterium aurantiacum* (Aa*_CrtZ*) was codon optimized, synthesized and cloned into pUC57-Simple	This study
pUC57-Simple-02	*CrtZ* from *Alcaligenes* sp. PC-1 (As*_CrtZ*) was codon optimized, synthesized and cloned into pUC57-Simple	This study
pUC57-Simple-03	*CrtZ* from *Erwinia uredovora* (Eu*_CrtZ*) was codon optimized, synthesized and cloned into pUC57-Simple	This study
pUC57-Simple-04	*CrtZ* from *Pantoea agglomerans* (Pa*_CrtZ*) was codon optimized, synthesized and cloned into pUC57-Simple	This study
pUC57-Simple-05	*CrtZ* from *Pantoea stewartii* (Ps*_CrtZ*) was codon optimized, synthesized and cloned into pUC57-Simple	This study
pUC57-Simple-06	*CrtZ* from *Sulfolobus solfataricus* P2 (Ss*_CrtZ*) was codon optimized, synthesized and cloned into pUC57-Simple	This study
pUC57-Simple-07	*CrtZ* from *Brevundimonas* sp. SD212 (B.SD*_CrtZ*) was codon optimized, synthesized and cloned into pUC57-Simple	This study
pUC57-Simple-08	*CrtZ* from *Brevundimonas vesicularis* DC263 (B.DC*_CrtZ*) was codon optimized, synthesized and cloned into pUC57-Simple	This study
pUC57-Simple-09	*CrtZ* from *Haematococcus pluvialis* (Hp*_CrtZ*) was codon optimized, synthesized and cloned into pUC57-Simple	This study
pUC57-Simple-10	*CCD2* from *Crocus* was codon optimized, synthesized and cloned into pUC57-Simple	This study
pUC57-Simple-11	*CCD3* from *Crocus* was codon optimized, synthesized and cloned into pUC57-Simple	This study
pUC57-Simple-12	*ZCD* from *Crocus* was codon optimized, synthesized and cloned into pUC57-Simple	This study
pUC57-Simple-13	*ZCD1* from *Crocus* was codon optimized, synthesized and cloned into pUC57-Simple	This study
pUC57-Simple-14	*ALD6* from *S. cerevisiae* was cloned into pUC57-Simple	This study
pUC57-Simple-15	*ALD* from *Bixa orellana* (Bix*_ALD*) was codon optimized, synthesized and cloned into pUC57-Simple	This study
pUC57-Simple-16	*ALD* from *Synechocystis* sp. PCC6803 (Syn*_ALD*) was codon optimized, synthesized and cloned into pUC57-Simple	This study
pJET1.2-Z-01	The cassette *ho_*F-P_*GAL1*_-T_*HIS5*_-*URA3*-*ho_*R was cloned and inserted into the pJET1.2	This study
pJET1.2-Z-02	Aa*_CrtZ* was digested from pUC57-Simple-01 by *Bsa*I and inserted into the same site of pJET1.2-Z-01	This study
pJET1.2-Z-03	As*_CrtZ* was digested from pUC57-Simple-02 by *Bsa*I and inserted into the same site of pJET1.2-Z-01	This study
pJET1.2-Z-04	Eu*_CrtZ* was digested from pUC57-Simple-03 by *Bsa*I and inserted into the same site of pJET1.2-Z-01	This study
pJET1.2-Z-05	Pa*_CrtZ* was digested from pUC57-Simple-04 by *Bsa*I and inserted into the same site of pJET1.2-Z-01	This study
pJET1.2-Z-06	Ps*_CrtZ* was digested from pUC57-Simple-05 by *Bsa*I and inserted into the same site of pJET1.2-Z-01	This study
pJET1.2-Z-07	Ss*_CrtZ* was digested from pUC57-Simple-06 by *Bsa*I and inserted into the Same site of pJET1.2-Z-01	This study
pJET1.2-Z-08	B.SD*_CrtZ* was digested from pUC57-Simple-07 by *Bsa*I and inserted into the Same site of pJET1.2-Z-01	This study
pJET1.2-Z-09	B.DC*_CrtZ* was digested from pUC57-Simple-08 by *Bsa*I and inserted into the Same site of pJET1.2-Z-01	This study
pJET1.2-Z-10	Hp*_CrtZ* was digested from pUC57-Simple-09 by *Bsa*I and inserted into the Same site of pJET1.2-Z-01	This study
pRS416-C-01	The cassette T_*HIS5*_-P_*GAL10*_-*CCD2*-T_*TEF2*_ was cloned and inserted into the *Not*I site of pRS416	This study
pRS416-C-02	The cassette T_*HIS5*_-P_*GAL10*_-*CCD3*-T_*TEF2*_ was cloned and inserted into the *Not*I site of pRS416	This study
pRS416-C-03	The cassette T_*HIS5*_-P_*GAL10*_-*ZCD*-T_*TEF2*_ was cloned and inserted into the *Not*I site of pRS416	This study
pRS416-C-04	The cassette T_*HIS5*_-P_*GAL10*_-*ZCD1*-T_*TEF2*_ was cloned and inserted into the *Not*I site of pRS416	This study
pRS425 K-A-01	The cassette T_*TEF2*_-P_*GAL7*_-T_*PGI1*_ was cloned and inserted into the *Pst*I/*Bam*HI site of pRS425 K	This study
pRS425 K-A-02	*ALD6* was digested from pUC57-Simple-14 by *Bsa*I and inserted into the same site of pRS425 K-A-01	This study
pRS425 K-A-03	Bix*_ALD* was digested from pUC57-Simple-15 by *Bsa*I and inserted into the same site of pRS425 K-A-01	This study
pRS425 K-A-04	Syn*_ALD* was digested from pUC57-Simple-16 by *Bsa*I and inserted into the same site of pRS425 K-A-01	This study
pRS416-A-01	The cassette T_*HIS5*_–T_*PGI1*_ was cloned and inserted into the *Xho*I/*Sac*I site of pRS416	This study
pRS416-A-02	The cassette T_*HIS5*_-P_*GAL10*_-*CCD2*-T_*TEF2*_ (digested from pRS416-C-01 by *Not*I), the cassette T_*TEF2*_-P_*GAL7*_-*ALD6*-T_*PGI1*_ (digested from pRS425 K-A-02 by *Pst*I/*Bam*HI) and plasmid pRS416-A-01 (digested by *Bam*HI) were assembled based on RADOM method	This study
pRS416-A-03	The cassette T_*HIS5*_-P_*GAL10*_-*CCD2*-T_*TEF2*_ (digested from pRS416-C-01 by *Not*I), the cassette T_*TEF2*_-P_*GAL7*_-Bix*_ALD*-T_*PGI1*_ (digested from pRS425 K-A-03 by *Pst*I/*Bam*HI) and plasmid pRS416-A-01 (digested by *Bam*HI) were assembled based on RADOM method	This study
pRS416-A-04	The cassette T_*HIS5*_-P_*GAL10*_-*CCD2*-T_*TEF2*_ (digested from pRS416-C-01 by *Not*I), the cassette T_*TEF2*_-P_*GAL7*_-Syn*_ALD*-T_*PGI1*_ (digested from pRS425 K-A-04 by *Pst*I/*Bam*HI) and plasmid pRS416-A-01 (digested by *Bam*HI) were assembled based on RADOM method	This study
pRS426-A-01	The cassette T_*HIS5*_–T_*PGI1*_ was cloned and inserted into the *Xho*I/*Sac*I site of pRS426	This study
pRS426-A-02	The cassette T_*HIS5*_-P_*GAL10*_-*CCD2*-T_*TEF2*_ (digested from pRS416-C-01 by *Not*I), the cassette T_*TEF2*_-P_*GAL7*_-Syn*_ALD*-T_*PGI1*_ (digested from pRS425 K-A-04 by *Pst*I/*Bam*HI) and plasmid pRS426-A-01 (digested by *Bam*HI) were assembled based on RADOM method	This study

## Methods

### Construction of plasmids and strains

Primers and plasmids used in this study were listed in Additional file 1: Table S1; Table 1, respectively. All the heterologous genes including c*rtZ*, *ccd*, and *ald* were codon optimized (Additional file 1: Table S2) and synthesized by GENEWIZ (Suzhou, China). All these genes were delivered as pUC57-simple serious plasmids (Table 1). Promoters (P_*GAL1*_, P_*GAL7*_ and P_*GAL10*_), terminators (T_*HIS5*_, T_*TEF2*_, and T_*PGI1*_) and integration homologous arms (*ho_*L and *ho_*R) were amplified from the genomic DNA of *S. cerevisiae* CEN.PK2-1C, as well as the auxotroph marker *URA3* was amplified from the plasmid pRS416. Cassette *ho_*L-P_*GAL1*_-T_*HIS5*_-*URA3*-*ho_*R was assembled by overlap extension PCR (OE-PCR) and cloned into pJET1.2, obtaining the plasmid pJET1.2-Z-01 (Table 1; Additional file 1: Figure S1). Genes *crtZ* were recovered by *Bsa*I digestion from pUC57-Simple-01–09 and inserted into the same site of pJET1.2-Z-01, generating pJET1.2-Z series plasmids (CrtZ expression cassette plasmids pJET1.2-Z-02–10, Table 1; Additional file 1: Figure S1). Then the CrtZ expression cassette *ho_*L-P_*GAL1*_-CrtZ-T_*HIS5*_-*URA3*-*ho_*R were cut from pJET1.2-Z series plasmids by *Pme*I and transformed into *S. cerevisiae* SyBE_SC0014CY06 for genomic integration (Fig. 1b) via the lithium acetate method [22]. Marker *URA3* was deleted according to Boeke et al. [23], obtaining zeaxanthin producing strains SyBE_Sc0123Cz10-18 (Table 1) as the host cell in our study.

For constructing the initial crocetin producing strain and screening CrtZ/CCD combination, only heterologous CCDs were carried by single copy plasmid pRS416 and introduced into zeaxanthin producing strains (Fig. 1b). Genes *ccd* were amplified from the plasmid pUC57-Simple-10–13 and assembled together with promoter P_*GAL10*_, terminators T_*HIS5*_ and T_*TEF2*_ into CCD expression cassette T_*HIS5*_-P_*GAL10*_-CCD-T_*TEF2*_ by OE-PCR. The products were inserted into the *Not*I site of plasmid pRS416, obtaining pRS416-C serious plasmids (CCD expression plasmids pRS416-C-01-04, Table 1; Additional file 1: Figure S2). These plasmids were transferred into zeaxanthin producing strains according to Table 1, producing crocetin producing strains (Table 1).

For screening ALD sources, heterologous CCD and ALD were carried by centromeric plasmid pRS416 and introduced into zeaxanthin producing strain (Fig. 1c). Cassette T_*TEF2*_-P_*GAL7*_-T_*PGI1*_ was also assembled by OE-PCR and cloned into pRS425 K, obtaining the plasmid pRS425 K-A-01 at first (Table 1; Additional file 1: Figure S3). Genes *ald* were recovered by *Bsa*I digestion from pUC57-Simple-14–16 and inserted into the same site of pRS425 K-A-01, generating pRS425 K-A series plasmids (pRS425 K-A-02–04, Table 1; Additional file 1: Figure S3). Meanwhile, cassette T_*TEF2*_-T_*PGI1*_ was assembled by OE-PCR. The product was incubated with *Xho*I/*Sac*I and inserted into the same sites of pRS416, producing pRS416-A-01. Then cassettes T_*HIS5*_-P_*GAL10*_-*ccd2*-T_*TEF2*_ (digested from pRS416-C-01 by *Not*I), T_*TEF2*_-P_*GAL7*_-*ald*-T_*PGI1*_ (digested from pRS425 K-A-02–04 by *Pst*I/*Bam*HI) and linearized vector pRS416-A-01 (digested by *Bam*HI) were assembled based on RADOM method in the particular zeaxanthin producing strain (producing strains SyBE_Sc0123C048–50 harboring plasmids pRS416-A-02–04 respectively, Table 1; Additional file 1: Figure S3) [24]. For adjusting the expression level of CCD and ALD, multiple plasmid pRS426, instead of pRS416, was employed to carry CCD and ALD expression cassettes. Similar procedures were taken as motioned above, which were presented in Additional file 1: Figure S3.

### Strains and culture conditions

*Escherichia coli* DH5α or TransT1 was used for plasmid construction, which was cultured at 37 °C in Luria–Bertani medium [15] supplemented with 50 μg/mL kanamycin or 100 μg/mL ampicillin for selection. Meanwhile, all the engineered yeast strains summarized in Table 1 were based on an existing β-carotene producing strain, *S. cerevisiae* SyBE_SC0014CY06. Engineered yeast strains were cultured on YPD medium or synthetic complete (SC) medium lacking appropriate nutrient component for selection [25]. When needed, 1% (w/v) d-(+)-galactose were used as the inducer in fermentations and supplied into YPD medium (generating YPDG medium).

For shake-flask cultivation, colonies on solid plates were picked up and cultured in 3 mL SC medium for overnight growth at 30 °C. Then the preculture was transferred into 25 mL fresh SC medium and grew until reaching to mid-log phase. After that, the seed culture was inoculated into 50 mL YPD medium with an initial OD600 of 0.1 and cultivated at 30 °C for 72 h or 20 °C for 96 h. All the fermentation experiments were performed in triplicate.

### Fed-batch fermentation

The strain SyBE_Sc0123C053 was used for fed-batch fermentation. 100 µL glycerol-stock was inoculated into 25 mL SC medium and cultured at 30 °C, 250 rpm for overnight growth. Then the preculture was transferred to 200 mL fresh SC medium and grew until entering mid-exponential phase. Seed cultures were transferred to 1.8 L YPD medium (20 g/L glucose) in a 5 L bioreactor (BLBIO-5GJG-2, Shanghai, China) at a 10% (v/v) inoculum. The pH was automatically controlled at 5.5 with ammonia hydroxide (6 M). And the dissolved oxygen was kept at 40% by agitation cascade from 400 to 600 rpm, while the air flow was set at 2.5 vvm.

As the crocetin production modules were controlled by employed galactose-inducible system, the fed-batch fermentation should be divided into two stages: cell growth stage and crocetin accumulation stage. During the period of the cell growth stage, fermentation was carried out at 30 °C. The glucose concentration was monitored every 2 h and the glucose consumption rate was obtained accordingly. Based on this data, the glucose concentration was maintained less than 1 g/L by adding an appropriate volume of concentrated glucose solution (500 g/L) continuously. And 5 g yeast extract was added into the bioreactor every 12 h by feeding 400 g/L yeast extract stock solution. When the cell growth fell into stable phase, fermentation entered the second stage: crocetin accumulation stage. Then after fermentation temperature reduced to 20 °C, 10 g/L of d-(+)-galactose was fed to induce crocetin biosynthesis. As glucose was exhausted, cells begun to use ethanol as carbon source. The ethanol concentration was controlled below 5 g/L through adjusting the feeding rate of ethanol until harvest. Duplicate samples were collected to determine the cell density, glucose concentration, ethanol concentration and crocetin production. To avoid the spontaneous degradation from light, bioreactor should be covered with foils.

### Extraction and analysis of carotenoids

To determine carotenoids accumulation, standards of lycopene, β-carotene and zeaxanthin were purchased from Sigma (Sigma-Aldrich, MO, USA), and standard of crocetin was purchased from Yuanye Bio-Technology (Shanghai, China). The procedures for extracting and analyzing carotenoids were modified according to Xie et al. [20]. To be specific, after harvested cells were washed with distilled water, the cell pellet was re-suspended in 3 N HCl and boiled for 2 min, and then immediately cooled in ice for 3 min. Then cells debris were harvested and resuspended in acetone containing 1% (w/v) butylated hydroxytoluene. The above mixture was vortexed until colorless. After centrifugation, the acetone phase containing the extracted carotenoid was collected and evaporated by nitrogen blow. The products were analyzed by high-performance liquid chromatography system (HPLC, Waterse2695, Waters Corp, USA) equipped with a BDS HYPERSIL C18 column (150 mm × 4.6 mm, 5 μm, Thermo Scientific) and a UV/VIS detector (Waters 2489). To characterize lycopene, β-carotene and zeaxanthin, the product was dissolved in acetone and the signals were detected at 450 nm. The mobile phase consisting of acetonitrile-methanol (65:35 v/v) was chosen with a flow rate of 0.8 mL/min and the column temperature was set at 25 °C. In the meanwhile, for crocetin analysis, sample was dissolved in methanol-dimethylformamide (7:1 v/v) and crocetin was detected at 430 nm. 70% (v/v) methanol–water (containing 2% formic acid) was utilized as the mobile phase with a flow rate of 1 mL/min at 40 °C. Notably, considering that carotenoids are extremely unstable and susceptible to light, brown centrifugal tubes were used in the above procedures to avoid exposure to light.

### Bioinformatics and structural analysis of CCD

The protein identified sequences of the target CCD from different taxa were queried from protein knowledgebase (UniProtKB) available at http://www.uniprot.org/, using the key term “carotenoid cleavage dioxygenase”, and subjected to a brief bioinformatics analysis to guarantee suitable diversity. Initially the CCD protein sequences were aligned by means of clustal W with default settings [26]. Phylogenetic tree of CCD gene family was conducted in MEGA7 [27] and inferred by Neighbor-Joining method [28]. The bootstrap consensus tree deduced from 1000 replicates was taken to represent the evolutionary history of the taxa analyzed [29].

The structures of the CCD2 and CCD3 were both modeled based on the target-template (PDB ID: 2biw) alignment using SWISS-MODEL [30, 31]. And the Coordinates which are conserved between the targets and the template are copied from the template to the model. Insertions and deletions are remodeled using a fragment library. Side chains are then rebuilt. Finally, the geometry of the resulting model is regularized by using a force field. The modeled structures of target proteins were resolved with PyMol software [32].

## Results and discussion

### Construction of inducible crocetin biosynthesis pathway

To realize crocetin biosynthesis, heterologous *crtZ* and *ccd* were codon optimized and introduced into an existing β-carotene producer (*S. cerevisiae* SyBE_SC0014CY06), which processed endogenous ALDs to catalyze the final step in crocetin synthesis pathway (Fig. 1a) [9]. At first, *crtZ* was integrated into the *ho* locus of the chromosome, while *ccd* was carried by centromeric plasmid pRS416. The expression of CrtZ and CCD were under the control of galactose-regulated GAL promoters GAL1 and GAL10, respectively (Fig. 1b). Because a highest zeaxanthin production was once achieved in yeast strain harboring CrtZ from *Erwinia uredovora* (Eu_CrtZ) among nine selected CrtZ species [33], Eu_CrtZ were also selected and intergraded into the chromosome of strain SyBE_SC0014CY06, generating strain SyBE_Sc0123Cz12 as a host cell in our study. In the meanwhile, CCD2 from *Crocus* was also selected to convert zeaxanthin to crocetin dialdehyde, obtaining strain SyBE_Sc0123C009. Strains SyBE_Sc0123C009 and SyBE_Sc0123Cz12 together with the parent strain SyBE_SC0014CY06 were cultured in shake-flask with YPDG medium at 30 °C and their products were analyzed by HPLC after 72 h incubation. As shown in Fig. 1d, crocetin (peak III) was successfully detected with a titer as 9.42 μg/L in strain SyBE_Sc0123C009, indicating that a functional crocetin biosynthesis pathway succeeded here. To be notably, there was no distinct β-carotene accumulation in zeaxanthin producing strain SyBE_Sc0123Cz12, while an amount of β-carotene (peak II), zeaxanthin (peak I), as well as other unidentified byproducts or intermediates were observed in crocetin producing strain SyBE_Sc0123C009 (Fig. 1e), suggesting that the step catalyzed by CCD was rate-limiting here and the selected CrtZ/CCD combination did not match well, which needed to be optimized further.

### Optimization of cultivation temperature

**Fig. 2:**
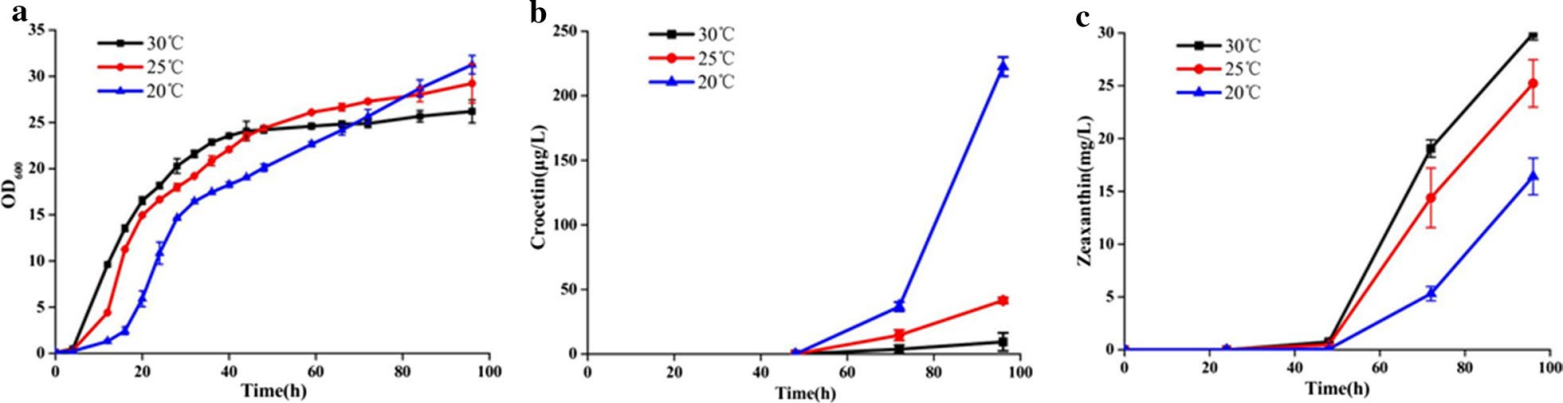
The effects of culture temperature on cell growth (**a**), crocetin production (**b**), and zeaxanthin accumulation (**c**). *S. cerevisiae* strain SyBE_Sc0123C009 was cultivated in YPDG media under different cultivation temperature (30 °C shown in *squares*, 25 °C in *circles* and 20 °C in *triangles*), respectively, in shake-flasks for analysis by HPLC. The* error bars* represent standard deviation calculated from triplicate experiments

It is reported by Shi et al. [34] that low temperature was benefit for carotenoids accumulation in *Phaffia rhodozyma.* In our study, by cultivation of series zeaxanthin producing strains at 20 and 30 °C respectively, it was also found that the production of zeaxanthin was higher at 20 °C than that at 30 °C (Additional file 1: Figure S4), indicating lower temperature benefited much for zeaxanthin production, which would provide more sufficient precursor supplies for higher crocetin production. Moreover, concerning that root development and flower emergence occur at low temperature for *Crocus* plants, and the expression of CCD were induced by low temperature in *Crocus* [35–37], the effect of culture temperature was also investigated here. Thus, for higher crocetin titer, the culture temperature for strain SyBE_Sc0123C009 was decreased from 30 °C, via 25 to 20 °C. The cell density, zeaxanthin accumulation and crocetin production were measured during the time course. As a result, in case of cell growth, there was a longer lag phase under lower temperature, compared to cultivating at 30 °C (Fig. 2a). Meanwhile, a dramatical increase on crocetin production along with a decrease on zeaxanthin accumulation was achieved by reducing cultivation temperature (Fig. 2b, c), suggesting 20 °C was the optimal temperature for converting zeaxanthin to crocetin. Javiera López et al. [38] once reported that β-ionone producing yeast strain processing CCD1, the homologue of CCD2, worked much better at low temperature, which showed similar results as our study. Finally, the crocetin titer reached 223 μg/L at 20 °C after 96 h fermentation in shake-flask (Fig. 2b). And 20 °C was used as the culture temperature in further study.

### Optimal CrtZ/CCD combination by screening enzymes from diverse sources

**Fig. 3:**
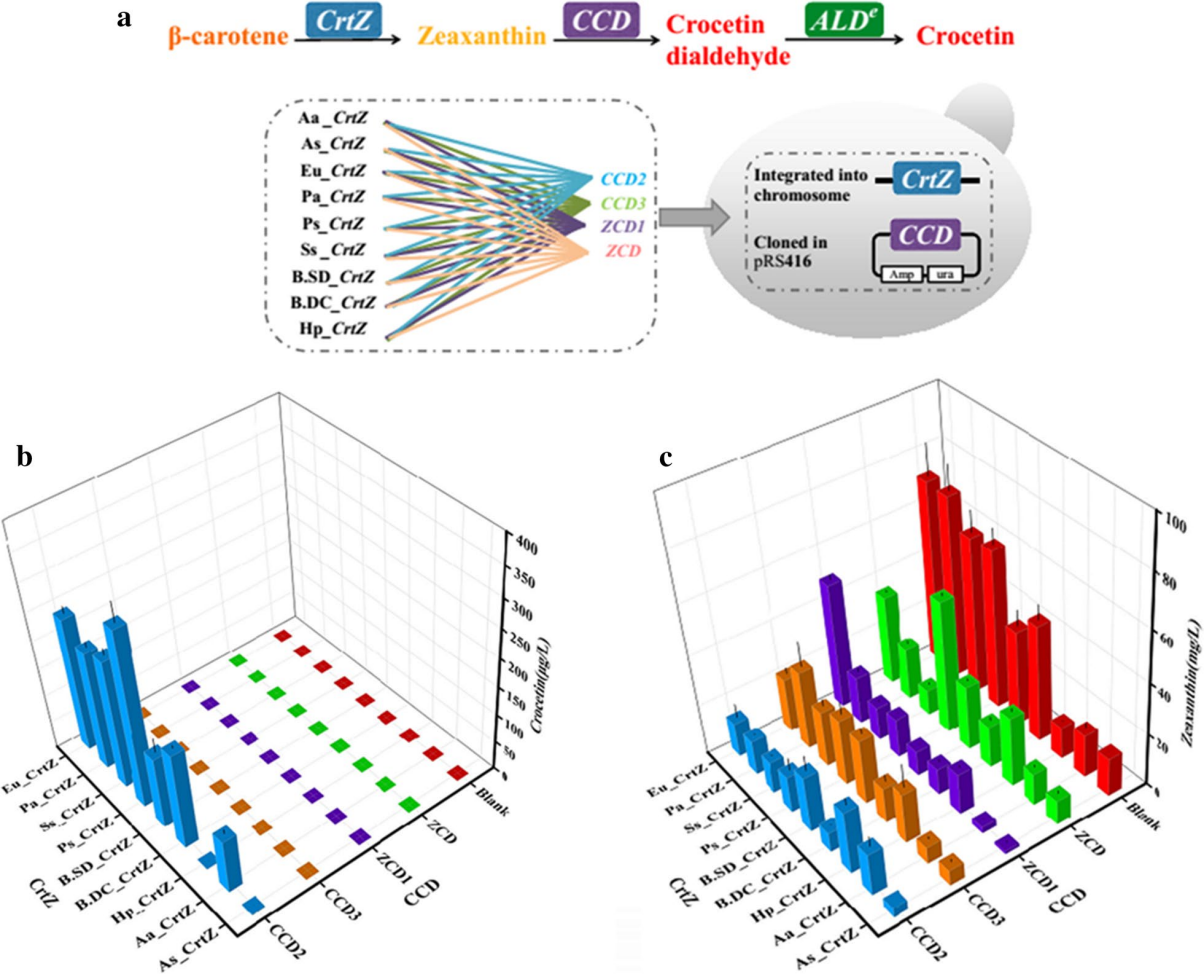
Combinatorial optimization of CrtZ and CCD from diverse species. **a** The sketch map of combinations. Each pair of CrtZ and CCD was connected by *solid lines* in specific color, forming a tested group. Consequently, 36 crocetin producing strains were constructed by screening enzymes from different sources. Eu, *E. uredovora*; Pa, *Pantoea agglomerans*; Ss, *Sulfolobus solfataricus* P2; Ps, *Pantoea stewartii*; B.SD, *Brevundimonas* sp. SD212; B.DC, *Brevundimonas vesicularis* DC263; Hp, *Haematococcus pluvialis*; Aa, *Agrobacterium aurantiacum*; As, *Alcaligenes* sp. PC-1. Determination of crocetin production (**b**) and zeaxanthin accumulation (**c**) in each combination by shake flask fermentation. The nine zeaxanthin producing strains carrying different CrtZs without CCDs were used as the blank control. The *error bars* represent standard deviations calculated from triplicate experiments

**Fig. 4:**
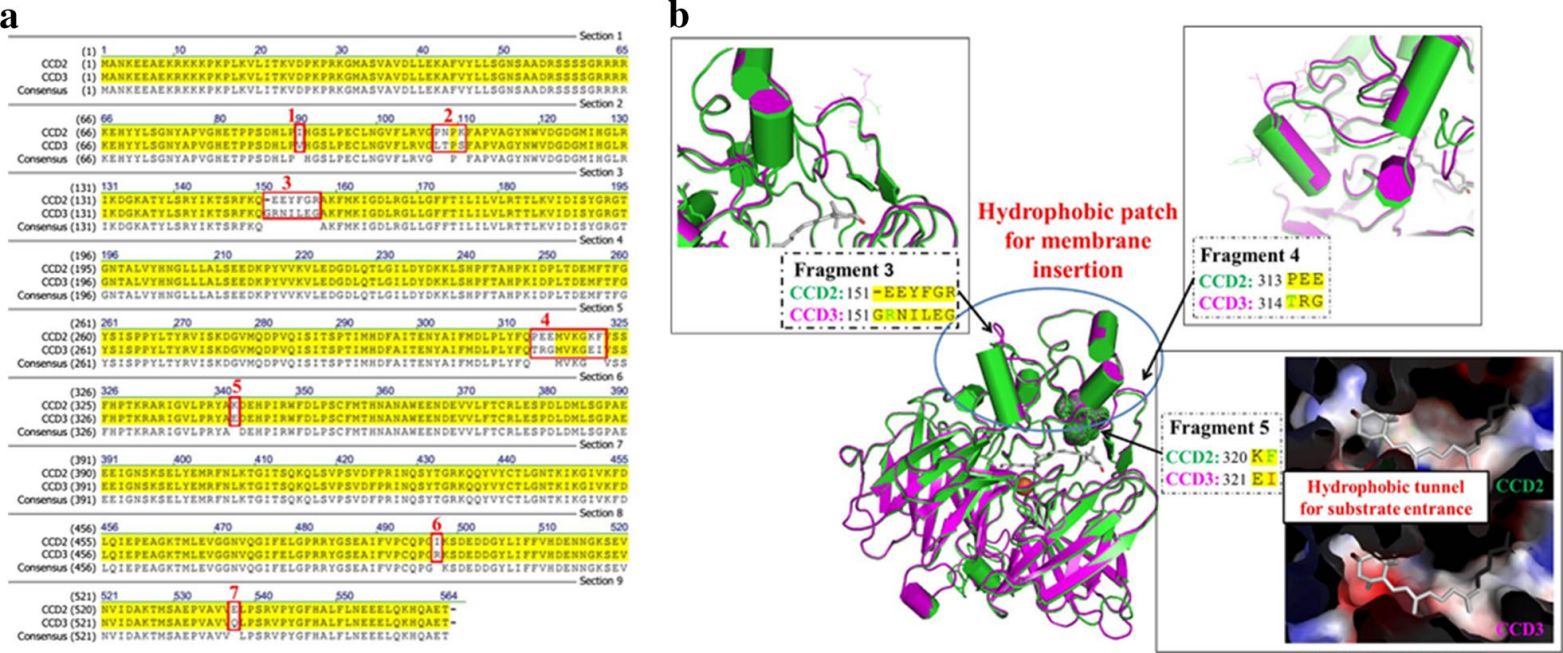
Sequences and architecture differences between CCD2 and CCD3. **a** Sequences alignment of CCD2 and CCD3. The dissimilar sequence fragments were *boxed with red lines*, and fragment numbers are indicated in the respective positions. **b** Alignment of structural models of CCD2 and CCD3. Fragments 3 and 4 show the difference structure in the hydrophobic patches for putative membrane insertion. Fragment 5 shows the difference protein contact potential in the hydrophobic tunnel for substrate entrance. CCD2 and CCD3 are colored in *green* and *purple*, respectively. Negative potential are *red*, positive potential are *blue* and neutral potential are *white*

As mentioned above, combinatorially screening enzymes from diverse sources has been proved to be a promising method to obtain the best combination in terms of substrate selectivity, catalytic activity and host cell compatibility, which would lead to higher productivity of the target compound [39–42]. Through blastp searching through NCBI database (https://blast.ncbi.nlm.nih.gov/Blast.cgi?PROGRAM=blastp&PAGE_TYPE=Blast) Search&LINK_LOC = blasthome based on the sequence of CCD2, CCD3 showed a 97% identity with CCD2 (Fig. 4a). Hence besides three crocetin synthesis related CCDs (ZCD, ZCD1 and CCD2) described before [10, 17, 18], CCD3 was selected as potential candidate in our study. Here, these four CCDs together with nine CrtZs from *E. uredovora* (Eu_CrtZ), *Pantoea agglomerans* (Pa_CrtZ), *Sulfolobus solfataricus* P2 (Ss_CrtZ), *Pantoea stewartii* (Ps_CrtZ), *Brevundimonas* sp. SD212 (B.SD_CrtZ), *Brevundimonas vesicularis* DC263 (B.DC_CrtZ), *Haematococcus pluvialis* (Hp_CrtZ), *Agrobacterium aurantiacum* (Aa_CrtZ), *Alcaligenes* sp. PC-1 (As_CrtZ) were expressed in strain SyBE_SC0014CY06, generating 36 strains with diverse CrtZ/CCD combinations (Fig. 3a; Table 1). Nine strains carrying different CrtZs without CCDs introduced were used as the blank control (Fig. 3b, c; Table 1). All the above strains were cultured in YPDG medium to analyze the accumulation of zeaxanthin and crocetin. As illustrated in Fig. 3b, only the strain harboring CCD2 instead of other three CCDs could achieve crocetin accumulation in yeast, furtherly demonstrating CCD was a rate-limiting enzyme in crocetin synthesis pathway. Rather than CrtZ, CCD seemed to be more crucial for crocetin production. Finally, the combination as Ps_CrtZ/CCD2 achieved the highest crocetin titer as 351 μg/L in strain SyBE_Sc0123C017. This optimal combination would be a promising candidate for further optimization.

In this study, ZCD, ZCD1 and CCD3 could not achieve crocetin production in yeast, which required sequential cleavage at C7–C8 and C7′–C8′ double bonds adjacent to the 3-OH-β-ionone ring [43]. Even though there was no crocetin detected in strains carrying these three enzymes separately, zeaxanthin accumulations were consumed at varying degrees in these strains, suggesting their cleave activities in yeast might at other position or only at one side of the molecules. Among the five subfamilies of plant CCDs, the CCD1 and CCD4 families were the only two involved in the cleave activities at 7, 8/7′, 8′ positions [44, 45]. A phylogenetic analysis of CCD sequences from diversity sources belonging to CCD1 and CCD4 families, illustrated that CCD2 and CCD3 belonged to CCD1 subfamily, while ZCD and ZCD1 were members of CCD4 subfamily (Additional file 1: Figure S5).

For CCD2 and CCD3, they shared 97% identities and exhibited dramatically diversity on enzyme activities. Through alignment of their protein sequences, seven dissimilar short fragments were detected (Fig. 4a). In order to further characterize these differences, the structural models of CCD2 and CCD3 were generated based on the crystal structure of their homological protein apocarotenoid cleavage oxygenase from *Synechocystis* (PDB accession ID: 2biw). As shown in Fig. 4b, CCD comprised seven bladed β-propellers, which is highly conserved among all CCDs and covered by a less rigid dome formed by a series of loops [46]. To be notably, there is a tunnel perpendicular to the propeller axis of CCD. As reported, the tunnel acted as a channel for the passage of their hydrophobic substrates to the active site, and was consisted of hydrophobic residues (mainly Phe, Val, Leu) interacting with their lipophilic substrates via hydrophobic forces to guarantee both the specificity and correct orientation of substrate for the cleavage reactions [47]. Thus, when the high hydrophobicity of the tunnel was subsided by the alteration in Fragment 5 (which located at the tunnel) as the residues of K320-F321 from CCD2 and E321-I322 from CCD3 (Fig. 4b), the substrate entrance to CCD3 was impacted for change on substrate specificity consequently. Meanwhile, the entrance of the tunnel located in a large hydrophobic patch for membrane insertion, which provided an appropriate environment for lipophilic substrates accommodation and enzyme contraction. The function of this hydrophobic patch mainly depended on the stable α-helices region, which was involved in the Fragment 3 and Fragment 4. The structure of CCD3 in Fragment 3 and Fragment 4 showed the longer and more unstable loops than that of CCD2 (Fig. 4b). As illustrated in Fig. 3c, besides substrates selectivity, CCD3 exhibited lower cleavage activity on zeaxanthin than CCD2, no matter cooperated with what kind of CrtZ sources. These results could be explained by above descriptions. Moreover, there were still some variances between CCD3 and CCD2 which could not support above results by current protein model. Therefore, a more delicate phylogenetic analysis of CCD sequences only from CCD1 family members were performed and showed that those unexplained different residues were highly conserved among all the tested CCD1 subfamily members except CCD3 (Additional file 1: Figure S7), suggesting the alternation on these conserved regions which might be essential to CCD function would reduce enzyme activities.

For ZCD and ZCD1, they share 96% identities (Additional file 1: Figure S6), and both truncated at the N-terminal as lacking a blade of β-propeller and part of the dome in classic CCD4 subfamily members. The truncation was once proved to lead to loss on any cleavage activity for ZCD in *E. coli* [18]. ZCD1 was reported to once achieve crocetin production in *C. vulgaris* [10]. However, in our study, both these two enzymes could not sequentially cleave zeaxanthin on 7, 8/(7′, 8′) positions in yeast (Fig. 3b). These conflicting dates highlight the importance of host cell compatibility on the performance of heterologous enzymes, which were also corroborated by the reports from Greene et al. [48].

### Screening ALD sources and fine-tuning of CCD/ALD

**Fig. 5:**
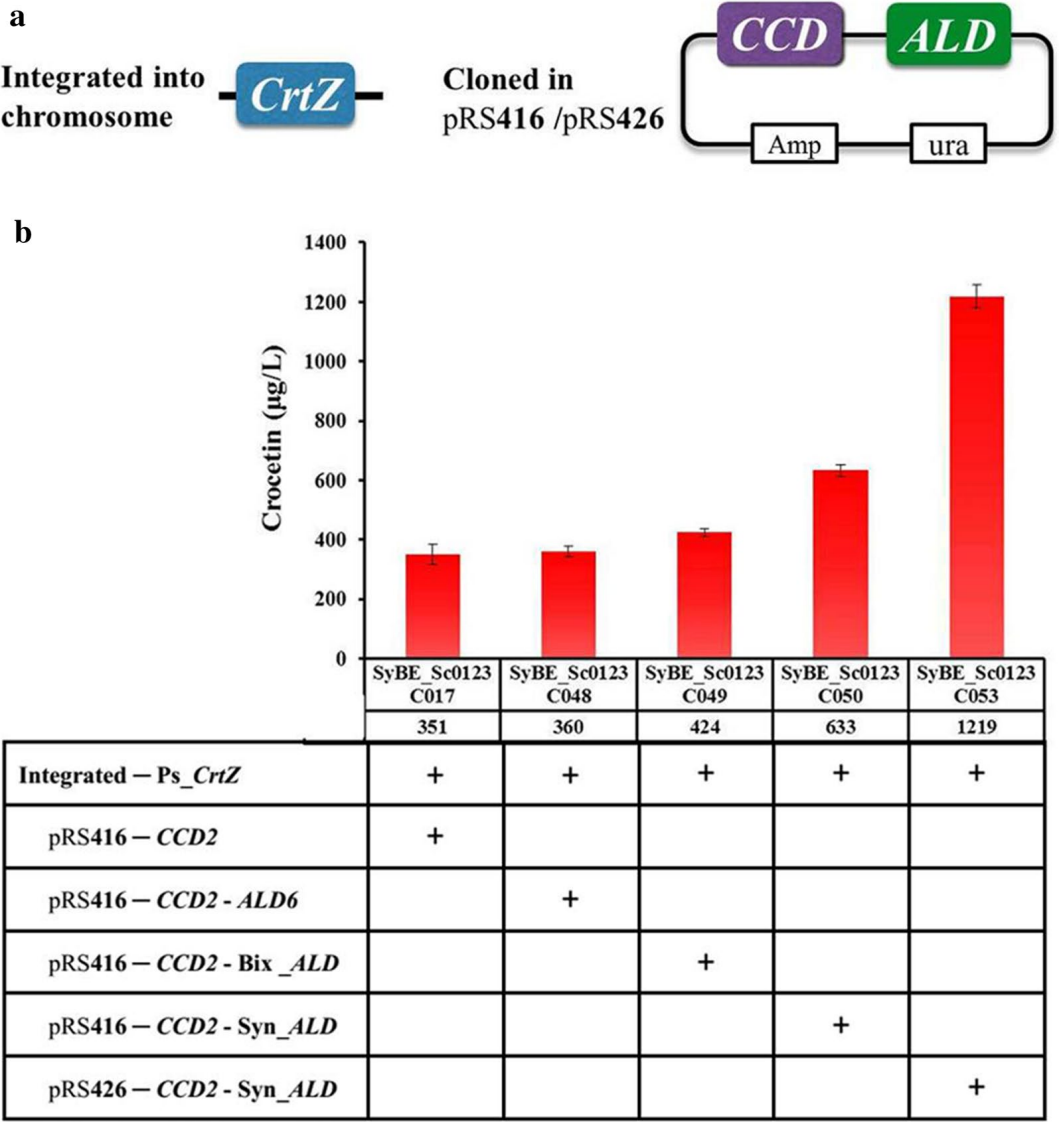
Screening ALD sources and fine-tuning of CCD/ALD. **a** The diagrammatic sketch representation of the engineering strategies for CrtZ, CCD and ALD expression modular. CrtZ expression cassette was integrated into the *ho* locus of the chromosome, CCD2 and ALD were carried in plasmid pRS416/pRS426. **b** The effect of ALD sources and the expression level of CCD/ALD on crocetin production. Ps, *Pantoea stewartii*; Bix, *Bixa orellana;* Syn, *Synechocystis* sp. PCC6803. The *error bars* represent standard deviation calculated from triplicate experiments

As so far, there is no ALD has been identified in *Crocus* for crocetin synthesis. Meanwhile, except endogenous ALDs in yeast (such as ALD6) and algae, none heterologous ALD has been reported yet to realize crocetin producing. Since the current crocetin titer, which was achieved by yeast endogenous ALDs, was still low, it is urgent to search and screen ALD isozymes from other organisms for higher crocetin production. Here, besides yeast endogenous ALD6 [49], two heterologous ALD originated from *Bixa orellana* (Bix_ALD) [50] and *Synechocystis* sp. PCC6803 (Syn_ALD) [51], whose substrates share the similar structure with crocetin dialdehyde, were selected and introduced together with CCD2 into the strain with Ps_CrtZ integrated in its chromosome (Fig. 5a). CCD2 and ALD were carried by single copy plasmid pRS416 and placed under the control of promoters GAL10 and GAL7, respectively (Fig. 1c). After growing in YPDG medium for 96 h, strain SyBE_Sc0123C050 harboring Syn_ALD achieved higher crocetin titer as 633 μg/L (Fig. 5b). Moreover, by increasing copy numbers of CCD2 and Syn_ALD via interchange of vector pRS416 into multicopy plasmid pRS426, the crocetin titer further improved to 1219 μg/L (Fig. 5b), obtaining strain SyBE_Sc0123C053 for bioreactor experiment.

### Optimization of crocetin production in bioreactor

**Fig. 6:**
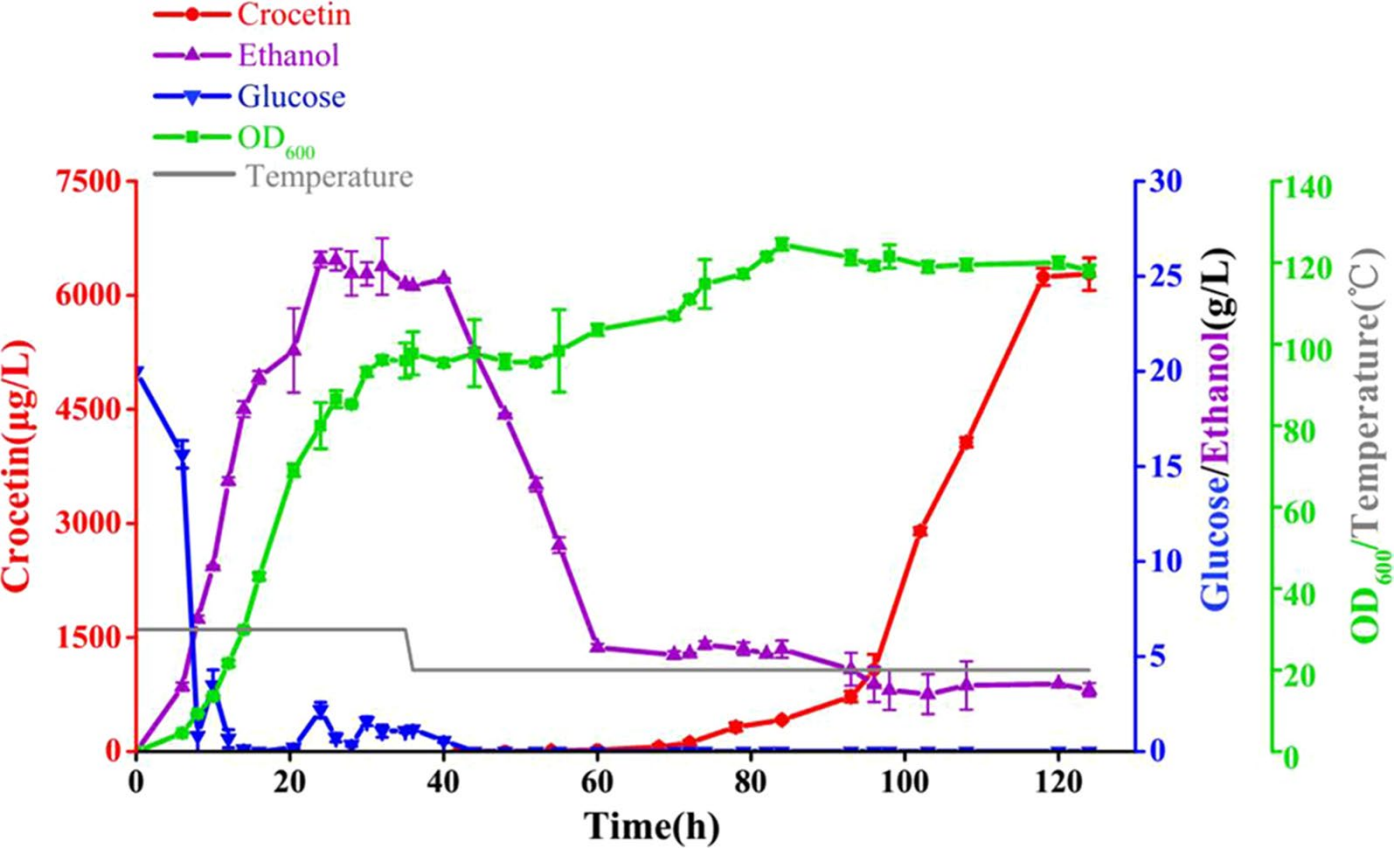
Crocetin production in fed-batch fermentation. Profile of crocetin production (*red line*), glucose (*blue line*), ethanol (*purple line*), cell density (*green line*) and fermentation temperature (*gray line*) in strain SyBE_Sc0123C053 during fed-batch fermentation. The *error bars* represent standard deviation calculated from duplicate experiments

To evaluate the production performance of the engineered strain SyBE_Sc0123C053, fed-batch fermentation was performed at a 2 L scale using YPD as the medium (Fig. 6). During cell growth stage, based on carbon restriction strategy, glucose concentration was strictly restricted. Cell density reached 96 for 35 h cultivation at 30 °C. There was also no acetate observed in this stage (data was not shown). When the culture temperature reduced to 20 °C at 36 h, d-(+)-galactose was added to induce crocetin production. After the initial ethanol generated by glucose was consumed below 5 g/L, additional 100 mL ethanol was fed into the medium periodically to maintain ethanol concentration around at 5 g/L until harvest. Eventually, a crocetin titer of 6278 μg/L was obtained after 124 h cultivation (Fig. 6), which was the highest reported titer in eukaryotic cell to date. However, the absolute titer (6278 μg/L) and the production yield based on ethanol consumption (Y_P/S_ = 0.012%) were far away from commercialization, strain engineering by metabolic engineering as well as synthetic biology and process innovation would be two basic but efficient aspects to promote crocetin output. In terms of strain engineering, increasing the catalysis activity of CCD and strain tolerance to product were the main challenges. Combinatorial engineering of *S. cerevisiae* and crocetin biosynthesis pathway in parallel would probably meet the demand [15]. As recent study in process optimization has demonstrated great potential in isoprene overproduction (up to 24 g/L) [52], we believe that crocetin production by our engineered strain would be further improved by continuous efforts in metabolic engineering, synthetic biology and fermentation optimization.

## Conclusions

In our study, crocetin biosynthesis pathway was successfully established in *S. cerevisiae* through incorporating heterologous CrtZ and CCD in an existing β-carotene producing strain. Then the effects of culture temperature, combination of CrtZ/CCD, ALD from different species, as well as the expression level of CCD and ALD on crocetin were investigated respectively. Compared to culturing at 30 °C, the crocetin accumulation performed much better at 20 °C. The accumulation of crocetin was further promoted by 2.8-fold by screening of CrtZ/CCD combination and ALD sources. Moreover, the crocetin titer was reached to 1219 μg/L by overexpression of *ccd* and *ald*. Consequently, the highest reported crocetin titer of 6278 μg/L was obtained in 5-L bioreactors. This study promotes the opportunities for industrialization of crocetin and crocin. This study also sets a good reference for microbial production of pharmaceuticals and chemicals in complex structure by fine-tuning multiple enzymes systematically.

## Additional file

**Additional file 1: Table S1.** Oligonucleotides used in this study. **Table S2.** The Codon-optimized sequences of *CrtZ*, *CCD* and *ALD* involved in this study. **Figure S1.** Schematic representation of the engineering strategies for CrtZ expression cassette. **Figure S2.** Schematic representation of the engineering strategies for CCD expression cassette. **Figure S3.** Schematic representation of the engineering strategies for ALD expression cassette. **Figure S4.** The effect of temperature on zeaxanthin production in zeaxanthin producing strains. **Figure S5.** Phylogenetic tree of CCD genes family was constructed and inferred by Neighbor-Joining method. **Figure S6.** Sequence alignment of ZCD and ZCD1 to identify the conserved region. **Figure S7.** Sequence alignment of CCD1 genes subfamily.

## Abbreviations

CrtZ: β-carotene hydroxylase
CCD: carotenoid cleavage dioxygenase
ALD: aldehyde dehydrogenase
*Crocus*: *Crocus sativus* L.
Eu: *E. uredovor*a
Pa: *Pantoea agglomerans*
Ss: *Sulfolobus solfataricus* P2
Ps: *Pantoea stewartii*
B.SD: *Brevundimonas* sp. SD212
B.DC: *Brevundimonas vesicularis* DC263
Hp: *Haematococcus pluvialis*
Aa: *Agrobacterium aurantiacum*
As: *Alcaligenes* sp. PC-1
Bix: *Bixa orellana*
Syn: *Synechocystis* sp. PCC6803
