# Supplementary material for: Heterologous biosynthesis and manipulation of crocetin in Saccharomyces cerevisiae
Source: Microb Cell Fact. 2017 Mar 29;16:54. doi: 10.1186/s12934-017-0665-1 (PMC5371240; doi:10.1186/s12934-017-0665-1)
Supplement: Supplementary file 1 — Additional file 1: Table S1. Oligonucleotides used in this study. Table S2. The Codon-optimized sequences of CrtZ, CCD and ALD involved in this study. Figure S1. Schematic representation of the engineering strategies for CrtZ expression cassette. Figure S2. Schematic representation of the engineering strategies for CCD expression cassette. Figure S3. Schematic representation of the engineering strategies for ALD expression cassette. Figure S4. The effect of temperature on zeaxanthin production in zeaxanthin producing strains. Figure S5. Phylogenetic tree of CCD genes family was constructed and inferred by Neighbor-Joining method. Figure S6. Sequence alignment of ZCD and ZCD1 to identify the conserved region. Figure S7. Sequence alignment of CCD1 genes subfamily. [file 12934_2017_665_MOESM1_ESM.docx]

**Supporting information**

**Heterologous biosynthesis and manipulation of crocetin in *Saccharomyces cerevisiae***

Fenghua Chai^a,b＃^,Ying Wang^a,b#^, Xueang Mei^a,b＃^, Mingdong Yao^a,b^, Yan Chen^a,b^, Hong Liu^a,b^, Wenhai Xiao^a,b^*, Yingjin Yuan^a,b^

^a^ Key Laboratory of Systems Bioengineering (Ministry of Education), Tianjin University, Tianjin, 300072, PR China.

^b^ SynBio Research Platform, Collaborative Innovation Center of Chemical Science and Engineering (Tianjin), School of Chemical Engineering and Technology, Tianjin University, Tianjin, 300072, PR China.

*Corresponding author:

Wenhai Xiao (Email: wenhai.xiao@tju.edu.cn, Tel: 86-22-60973987, Postal address: No. 92, Weijin Road, Nankai District, Tianjin, 300072, PR China)

# These authors contributed equally to this work

**Supplementary Table S1.** Oligonucleotides used in this study

| **Oligo name** | **Sequence (5’- 3’)** |
| --- | --- |
| **For construction of cassette *ho*_F-P*_GAL1_*-T*_HIS5_*-*URA3*-*ho*_R** | |
| *ho*-left-F^a^ | *GTTTAAAC*AACAGAATGAGTGGTATTAAAG |
| *ho*-left-R^b^ | GAATTTTTGAAAATTCAATATAAACGAAATGAACATAAATATACGGTG |
| GAL1p_F^b^ | TATATTTATGTTCATTTCGTTTATATTGAATTTTCAAAAATTC |
| GAL1p_R^a,b^ | CTATTTTAGGAGACC*GGTCTCC*CATTTATAGTTTTTTCTCCTTGAC |
| HIS5t-F^a,b^ | TATAAATGGGAGACC*GGTCTCC*TAAAATAGATTAATTTAAACAGTATATG |
| HIS5t-R^b^ | AAAATGATGAATTGAATTGAAAAATTCATCCTCTATCATAG |
| URA3-F^b^ | TGATAGAGGATGAATTTTTCAATTCAATTCATCATTTT |
| URA3-R^b^ | TGCCCACATGGTCGGTGAGGGTAATAACTGATATAAT |
| *ho*-right-F^b^ | TATATCAGTTATTACCCTGGGAAAACCCTGGCGTTACC |
| *ho*-right-R^a^ | *GTTTAAAC*GTTTAAACATTACTGATACC |
| **For construction of cassette T*_HIS5_-*P*_GAL10_-CCD-*T*_TEF2_*** | |
| HIS5t-F^a^ | *GCGGCCGC*ATAGATTAATTTAAACAG |
| HIS5t-R^b^ | GTCAAGGAGAAAAAACTATAAAATTCATCCTCTATCATAG |
| GAL10p_F^b^ | ATGATAGAGGATGAATTTTATAGTTTTTTCTCCTTGACG |
| GAL10p_R | TTATATTGAATTTTCAAAAATTC |
| CCD2-F^b^ | GAATTTTTGAAAATTCAATATAAATGGCTAACAAGGAAGAAGCC |
| CCD2-R^b^ | GGAAGCAATAATTATTACTCTTAGGTTTCGGCTTGATGC |
| CCD3-F^b^ | GAATTTTTGAAAATTCAATATAAATGGCAAATAAGGAAGAAGCC |
| CCD3-R^b^ | GGAAGCAATAATTATTACTCTTAAGTTTCTGCTTGGTGTT |
| ZCD1-F^b^ | GAATTTTTGAAAATTCAATATAAATGCAAGTTGATCCAACTAAAG |
| ZCD1-R^b^ | GGAAGCAATAATTATTACTCTTATTGTTGAGACAACAATTC |
| ZCD-F^b^ | GAATTTTTGAAAATTCAATATAAATGCAGGTGGACCCAACCAAG |
| ZCD-R^b^ | GGAAGCAATAATTATTACTCTGGCAGCGAATATGGAGAGG |
| TEF2t-F | GAGTAATAATTATTGCTTCC |
| TEF2t-R^a^ | *GCGGCCGC*GGGGTAGCGACGGATTAATGGC |
| **For construction of cassette T*_TEF2_-*P*_GAL7_-*T*_PGI1_*** | |
| TEF2t-F^a^ | *GGATCC*GAGTAATAATTATTGCTTCC |
| TEF2t-R^b^ | AGGATAGTAAGCTGGCAAAGGGGTAGCGACGGATTAATG |
| GAL7p_F^b^ | CATTAATCCGTCGCTACCCCTTTGCCAGCTTACTATCCTTC |
| GAL7p_R^a,b^ | TTGTTTTAGGAGACC*GGTCTCC*CATTTTTTGAGGGAATATTC |
| PGI1t-F^a,b^ | AAAAATGGGAGACC*GGTCTCC*TAAAACAAATCGCTCTTAAATAT |
| PGI1t-R^a^ | *CTGCAG*GTAGTTTAGTGTTTTTCTTCC |
| **For construction of cassette T*_HIS5_-*T*_PGI1_*** | |
| HIS5t-F^a^ | *CTCGAG*ATAGATTAATTTAAACAGTATATGTAC |
| HIS5t-R^a,b^ | AGAGCGATTTGT*GGATCC*AAATTCATCCTCTATCATAG |
| PGI1t-F^a,b^ | GAGGATGAATTT*GGATCC*ACAAATCGCTCTTAAATATAT |
| PGI1t-R^a^ | *CGAGCTC*GTAGTTTAGTGTTTTTCTTCC |

^a^ Restriction site was in italic.

^b^ Homologous overhang-nucleotides were underlined

**Supplementary Table S2.** The Codon-optimized sequences of *CrtZ*, *CCD* and *ALD* involved in this study

| **Protein** | **Encoding sequences** |
| --- | --- |
| *CrtZ* from *Agrobacterium aurantiacum*  (Aa_*CrtZ*) | atgactaacttcttgatcgttgttgctactgttttggttatggaattgactgcttactctgttcacagatggatcatgcacggtccattgggttggggttggcacaagtctcaccacgaagaacacgaccacgctttggaaaagaacgacttgtacggtttggttttcgctgttatcgctactgttttgttcactgttggttggatctgggctccagttttgtggtggatcgctttgggtatgactgtttacggtttgatctacttcgttttgcacgacggtttggttcaccaaagatggccattcagatacatcccaagaaagggttacgctagaagattgtaccaagctcacagattgcaccacgctgttgaaggtagagaccactgtgtttctttcggtttcatctacgctccaccagttgacaagttgaagcaagacttgaagatgtctggtgttttgagagctgaagctcaagaaagaacttaa |
| *CrtZ* from *Alcaligenes* sp. PC-1  (As_*CrtZ*) | atgacacaatttttgatagttgtcgcaaccgtcttggtaatggaattaactgcctattccgttcatagatggatcatgcacggtccattaggttggggttggcataaaagtcatcacgaagaacatgatcacgccttggaaaagaatgacttgtacggtgtagttttcgctgttttggcaactatcttattcacagttggtgcttactggtggcctgtcttgtggtggattgcattgggtatgaccgtttacggtttgatttacttcatcttgcatgatggtttagtccaccaaagatggccattcagatacatacctagaagaggttacttcagaagattgtaccaagcccatagattacatcacgctgtagaaggtagagatcattgtgtttcttttggtttcatctacgcaccacctgtcgataaattgaagcaagacttaaagagatcaggtgtattaagaccacaagacgaaagaccttcataa |
| *CrtZ* from *Erwinia uredovora*  (Eu_*CrtZ*) | atgttgtggatttggaatgctttgatcgttttcgttaccgtcattggtatggaagttattgctgctttggctcataagtacattatgcatggttggggttggggttggcatttgtctcatcatgaacctagaaaaggtgcctttgaagttaatgacttgtacgctgttgttttcgctgccttgtctattttgttgatctacttgggttctactggtatgtggccattgcaatggattggtgctggtatgactgcttatggtttgttgtacttcatggttcacgatggtttggttcatcaaagatggccattcagatatatcccaagaaagggttacttgaagagactttacatggcccatagaatgcatcatgctgttagaggtaaagaaggttgcgtttcttttggtttcttgtatgctccaccattgtctaagttgcaagctactttgagagaaagacatggtgctagagctggtgctgctagagatgctcaaggtggtgaagatgacccagcttctggtaagtaa |
| *CrtZ* from *Pantoea agglomerans*  (Pa_*CrtZ*) | atgttggttaattctttgatagtaatcttgtcagttatagctatggaaggtatcgctgcatttacccatagatatattatgcacggttggggttggagatggcatgaatctcatcacactcctagaaaaggtgtcttcgaattgaacgatttatttgccgttgtcttcgctggtgttgctatcgcattaattgcagtcggtactgccggtgtatggccattgcaatggatcggttgtggtatgacagtttacggtttgttatactttttagttcatgacggtttggtccaccaaagatggccattccattggatacctagaagaggttatttgaagagattgtacgttgcacacagattacatcacgctgtaagaggtagagaaggttgcgtttcctttggtttcatttacgctagaaagccagctgatttgcaagccatattgagagaaagacatggtagaccacctaaaagagacgccgctaaggatagaccagacgcagcctcaccttcttcatccagtccagaataa |
| *CrtZ* from *Pantoea stewartii*  (Ps_ *CrtZ*) | atgttgtggatttggaatgccttaatagttttcgtcactgttgtcggtatggaagtagttgctgcattggcacataaatatataatgcatggttggggttggggttggcatttatcacatcacgaaccaagaaagggtgcttttgaagttaacgacttgtatgctgtcgtattcgcaattgttagtatagctttgatctactttggttctactggtatctggcctttacaatggattggtgccggtatgacagcttatggtttgttatacttcatggtacatgatggtttagttcaccaaagatggccattcagatacatacctagaaagggttatttgaagagattgtacatggcacatagaatgcatcacgccgtccgtggtaaagaaggttgtgtttcatttggtttcttgtacgctccacctttgtccaagttacaagcaaccttaagagaaagacacgccgctagatcaggtgcagccagagatgaacaagacggtgttgatacttcttcatccggtaaataa |
| *CrtZ* from *Sulfolobus solfataricus* P2  (Ss_CtrZ) | atgatgttaatttattacgttggtatggccgttttgacttttgtcggtatggaatttgtcgctagattgatgcataagtacgtaatgcacggtttgttatggttcatccatgaagatcatcacaaggaaaagcaagcagaattggaaaagaatgatttgttcggtttagttttcgcctccgttagtgtctatttgtttttcttaggtattcaaggttcttacgtcgctttgtcaatagcaatcggcatgtcttcttatggtatcgcttactttttcatacatgatatggttatccacgacagacatttgcacttaagatcatggggtttgaaacatagaccattcaaggatttgatcttggttcatgacattcatcacaaagaaggcaagggtaactggggtttcttgttcgtaatcaagggtttagataaggttcctatcttgaaagacgaataa |
| *CrtZ* from *Brevundimonas* sp. SD212  (B.SD_*CrtZ*) | atggcttggttgacttggattgccttgttcttaacagcatttttgggtatggaagccttcgcttggataatgcatagatatgtcatgcacggtttcttgtggtcctggcatagaagtcatcacgaaccacatgatcacccattggaaaagaatgacttattcgcagttgtctttgctgcaccagcaatcgtaatggttgccgtcggtttgcatttatggccttgggccttgcctgttggtttaggtattacagcttatggtatggtatactttttctttcatgatggtttagttcacagaagattccctaccggtttttctggtagatcaggtttctggactagaagaatacaagctcatagattgcatcacgcagtaagaaccagagaaggttgtgtttctttcggtttcttgtgggtcagatcagcaagagccttgaaagctgaattagcacaaaagagaggttcttcatccagtggtgcttaa |
| *CrtZ* from *Brevundimonas vesicularis* DC263 (B.DC_*CrtZ*) | atgtcatggccaactatgatcttgttattcttagctacatttttgggtatggaagtctttgcctgggctatgcatagatatgtaatgcacggtttgttatggacttggcatagatcccatcacgaaccacacgatgacgttttagaaagaaatgatttgttcgccgttgtctttgctgcacctgccattatattagtcgctttgggtttacatttgtggccttggatgttacctattggtttgggtgtaacagcttatggtttggtttactttttctttcatgacggtttggttcacagaagattccctaccggtatcgctggtagatcagcattttggactagaagaatacaagcacatagattacatcacgccgtcagaaccagagaaggttgtgtaagtttcggtttcttgtgggttagatcagcaagagccttaaaagctgaattgtctcaaaagagaggttcttcatccaacggtgcataa |
| *CrtZ* from *Haematococcus pluvialis*  (Hp_*CrtZ*) | atgttgtccaagttgcaatccatctccgttaaggctagaagagttgaattggctagagacatcaccagaccaaaagtttgcttacacgctcagagatgctctttggtcagattgagagttgcagctccacaaacagaagaagcagttggaactcaacaagcagcaggagcaggagacgaacattcagcagacgttgctttgcaacaattggatagagctatcgcagaaagaagagctagaaggaagagagaacaattgtcttaccaagccgctgctattgcagcttctattggagtttcaggtattgctatcttcgctacctacttgagatttgctatgcatatgacagttggaggagcagttccttggggagaagttgcaggtccattattattggtggttggaggtcaattgggtatggaaatgtacgctagatacgctcataaggctatttggcacgaatctccattaggttggctattgcacaagtctcatcatactccaagaaccggtccatttgaagctaacgatttgttcgctatcatcaagggtctaccagctatgttgttgtgtactttcggcttttggttgccaaacgttttaggtacagcttgtttcggagcaggattaggcattactttatacggcatggcttacatgttcgttcacgacggtttagtttccagaagatttccaacaggtccaattgcaggtttaccatacatgaagaggttgacagttgctcaccaattgcatcattcaggaaaatacggtggagcaccttggggaatgtttttaggtccacaagaattgcaacatattccaggagcagcagaagaagttgaaagattggtgttggagttggattggtctaagaggtaa |
| *CCD2* from *Crocus* | atggctaacaaggaagaagccgaaaagagaaagaagaagccaaagccattgaaagtcttgatcaccaaggtcgatccaaaaccaagaaagggaatggcttcagttgcagttgatttgttggaaaaggccttcgtttacttgttatccggtaattctgcagccgatagatcttcctcttctggtagaagaagaagaaaggagcactactacttgtcaggaaattacgctccagttggtcatgaaactccaccatcagatcatttgccaatccatggtagtcttcctgagtgtttaaacggagtcttcttgagagttggcccaaatccaaaattcgctccagtagcaggttataattgggttgatggagacggaatgattcacggtttaagaatcaaggacggtaaagctacctacttgtctagatacatcaagacctccaggttcaagcaagaagaatacttcggtagagccaagttcatgaagattggcgatttgagaggactattgggtttcttcaccatcttgatcttggtcttgagaaccaccttgaaggttatcgatatctcctacggtagaggtacaggtaatacagctttggtttaccataacggcttgttgttggctttgtcagaagaagacaagccatacgttgttaaggtgttggaagacggagatttgcaaactttgggtatcctagactacgacaagaagttgtcccatccttttactgctcatccaaaaatcgatccattgacagacgagatgttcactttcggttactctatctctccaccatacttgacctacagagttatctctaaggacggcgttatgcaagatccagttcaaatttccatcacctctccaactatcatgcacgatttcgctatcaccgaaaactacgctatcttcatggacttgccactatacttccaaccagaagaaatggtcaagggcaaattcgtttcctctttccatccaaccaagagagctagaattggagttttgccaagatacgctaaagacgaacatccaattaggtggttcgacttgccttcttgtttcatgacccataacgctaacgcttgggaagaaaacgacgaagttgttctattcacttgcagattggaatccccagatctagatatgttgtcaggtccagcagaagaagaaatcggtaatagcaagtccgagttgtacgaaatgaggttcaacttgaagaccggtattacctctcaaaagcaattgagcgttccatcagtcgatttcccaagaattaaccagtcctacacaggtagaaagcaacaatacgtctactgcactttgggtaacactaagatcaagggcatcgttaagttcgacttgcaaatcgaaccagaagcaggtaaaactatgttggaagttggaggtaacgttcaaggcattttcgaattgggtccaagaagatacggttcagaagctatcttcgtcccttgtcaaccaggaattaaatcagacgaagacgacggttacttgattttcttcgttcacgacgaaaacaacggtaaaagcgaagtcaacgtcattgacgctaagactatgtcagcagaaccagttgcagttgttgaattgccatctagagttccatacggttttcacgcattgttcttgaacgaagaagagttgcaaaagcatcaagccgaaacctaa |
| *CCD3* from *Crocus* | atggcaaataaggaagaagccgaaaagagaaagaagaaaccaaagcctttgaaagtcttaattacaaaggtagacccaaagcctagaaagggtatggcatctgttgccgtcgatttgttggaaaaggctttcgtttatttgttgtccggtaattctgctgctgatagatcttcatccagtggtagaagaagaagaaaggaacattactacttatctggtaactacgcaccagtcggtcacgaaactccaccttctgatcatttgccagtacacggttcattacctgaatgtttgaatggtgttttcttgagagtcggtttgacaccatctttcgctcctgtcgcaggttataactgggtagatggtgacggtatgatccatggtttaagaattaaggatggtaaagccacttatttgtctagatacatcaagacatcaagattcaaacaaggtagaaacatcttggaaggtgctaagttcatgaaaatcggtgacttgagaggtttgttgggtttctttacaatattgatcttggttttgagaactacattgaaagtcatcgatatttcatatggtagaggtaccggtaatactgcattagtttaccataacggtttgttattggccttgtccgaagaagataagccatatgttgtcaaagttttggaagacggtgacttgcaaaccttgggtatattggattacgacaagaaattgtcacatccttttactgctcacccaaagattgatcctttgacagacgaaatgtttaccttcggttattccattagtccaccttatttgacttacagagttatatctaaggatggtgttatgcaagacccagtccaaatatctatcacatcacctaccatcatgcatgatttcgctatcacagaaaactacgcaatttttatggacttgccattatacttccaaacccgtggtatggtaaagggtgaaatcgtttcttcattccacccaactaaaagagccagaattggtgttttacctagatacgctgaagatgaacatccaattagatggtttgacttgccttcttgtttcatgacacacaatgccaacgcttgggaagaaaacgatgaagtagttttgtttacttgtagattggaatccccagatttggacatgttgagtggtcctgctgaagaagaaattggtaattctaagtcagaattgtacgaaatgagattcaatttgaagactggtatcacaagtcaaaagcaattgtccgtcccaagtgtagatttccctagaattaatcaatcttacaccggtagaaagcaacaatacgtttactgtaccttgggtaacactaagattaaaggtatcgtcaagttcgatttgcaaattgaaccagaagcaggtaaaactatgttggaagtaggtggtaatgttcaaggtatatttgaattaggtcctagaagatatggttcagaagccatcttcgttccatgccaacctggtagaaaatccgatgaagatgacggttacttgatctttttcgttcatgacgaaaacaacggtaaatccgaagtaaacgttatcgatgcaaagacaatgagtgccgaaccagtagctgtcgtacaattaccatctagagttccttatggttttcatgctttgttcttaaacgaagaagaattgcaaaaacaccaagcagaaacttaa |
| *ZCD* from *Crocus* | atgcaggtggacccaaccaaggggatcggcctggcgaacacaagcttgcaattctccaacggtcgactccacgcgctatgcgagtatgacctcccctacgtcgttcgcctctccccggaagacggggacatctcaaccgtcggacgcatcgaaaacaacgtctccacaaaaagtacgaccgcccacccgaagacggatcccgtcaccggagagacgttcagcttcagctacggacccatccagccctacgtcacgtactcccgttacgactgcgacggcaagaaatcgggccccgacgtgcccatcttctctttcaaggaaccgtctttcgtccacgacttcgcgatcaccgaacactatgccgtcttccccgacatccagatcgtgatgaagccggccgagatcgttcgggggcgccgcatgatcggcccggacctcgagaaggtcccgaggctggggttgcttccccggtacgccacgtcggactccgagatgcgatggttcgacgtgccggggttcaacatggtacacgtggtgaacgcgtgggaggaggaaggcggggaggtcgtggtgatcgtggcgcccaacgtgagcccgatagagaacgccatcgaccggttcgacctcctccacgtgtcggtggagatggcgaggatcgagctcaagagcgggagcgtgtcgcggacgcttctctcggcggagaatctggatttcggggtgattcaccggggctattcggggaggaagagccggtatgcttacctcggagtcggggatccgatgccgaagattcgcggggtggtgaaggtggacttcgagctggccgggagaggggaatgcgtggtggcgaggagggagttcggcgtgggatgtttcggaggagagccgttctttgtgccggcatcatcgaagaagagcggaggcgaggaagacgatgggtacgtagtgagttacttgcatgacgagggaaagggagagtcgagtttcgtggtgatggatgcgcggtcgccggagctagagatcttggcggaggtggttctgccgcggcgagtaccgtacgggtttcatggcctcttcgttacggaagctgagctgctgtcacagcagtagggacgacaccgtaccactgaatgataaaagatatccacctgaacgtataatcatcacctctccatattcgctgcca |
| *ZCD1* from *Crocus* | atgcaagttgatccaactaaaggtatcggtttggctaacacatcattacaattttctaacggtagattgcatgcattgtgtgaatacgatttgccatacgttgttagattatcaccagaagatggtgacatttctactgttggtagaatcgaaaacaatgtttcaactaagtctactacagctcatccaaaaacagatccagttactggtgaaacattttctttttcatacggtccaatccaaccatacgttacttactcaagatacgatagacatggtaaaaagtctggtccagatgttccaattttctcttttaaagaaccatctttcgttcatgatttcgctatcacagatcattacgcagttttcccagatatccaaatcgttatgaagccagcagaaatcgttagaggtagaagaatgattggtccagatttggaaaaagttccaagattaggtttgttaccaagatatgctacttctgattcagaaatgagatggtttgatgttccaggttttaatatggttcatgttgttaacgcatgggaagaagaaggtggtgaagttgttgttattgttgctccaaatgtttcaccaatcgaaaacgcaatcgatagattggatttgatccatgtttctgttgaaatggctagagttgatttgagatctggttcagtttctagaacattgttgtctgctgaaaatttggatttcggtgttattcatagaggttactcaggtagaaaatctagatatgcttacttgggtgttggtgacccaatgccaaagatcagaggtgttgttaaggttgatttcgaattggctggtcgtggtgaatgtgttgttgcaagaagagaatttggtgttggttgttttggtggtgaaccatttttcgttccagcatcagaaggttctggtggtgaagaagatgatggttacgttgtttcatatttgcatgatgagggtaaaggcgaatcttcatttgttgttatggatgctagatctccagaattggaagttgttgcagaagttgttttaccaagaagagttccatacggtttccatggtttattcgttacagaagctgaattgttgtctcaacaataa |
| *ALD* from *Bixa orellana*  (Bix_*ALD*) | atggaatctactggtagagctccattgggtaatggtagaatcggtaacccaggtggtttggttgtccctgaaattaagtttactaagttattcattaacggtagattcgtagataacgtttcaggtcaaactttcgatacaagagatccaagaaccggtgacgttttagccactatagccgaagctgataaagatgacttggacttagcagttaagaatgcaagagaagcctttgatcatggtccatggcctagaatgtccggttacgaaagagctagaataatgactaagtttgccgatttggttgaagctttcatagaagaattggctgcattagatacagcagacgccggtaaaaccttaagtatgggtaaagctgtcgatattccagccgctgtacaaataatgagatattacgcaggtgcagccgataaattgcatggtcaaccattgaagatgaaccaagatactcaaggttacacattgaaagaccctattggtgtagttggtcacattataccatggaactaccctacaatgatgtttttcttgaaaaacgctccagcattagctgctggttgtaccatggtcgtaaagccagccgaacaaactcctttgtccgctttattttacgctcatttggcaagaatggccggtgttcctgatggtgttgtcaatgtagttccaggttttggtcctacagctggtgccgctgtttcttcacacatggatgtcgactccgtaagtttcaccggttcccaagaagttggtagagctattatgagagcagccgctcaatctaatttgaaaaacgtctcattggaattaggtggtaaaagtccagtaatcatttttgatgacgcagatgttgacatggccgtctctttgtcacaattggcttgtttcacaaataagggtgaaatttgcgttgcaacctcaagagtctatgtacaagaaggtatatacgatgctttggttaagaaaattgttgaagcagccagagaatggagatttgatactaaagcaaacatgggtccacaagttgacaaaaagcaattcgaaagagtcttgaagtacatcgatttgggtaaaagagaaggtgctaccttgttaactggtggtaaaactaatggtgacaagggttactacatccaaccaacaatctttttggatgttagagaagacatgaacatagcacaagatgaattgtttggtcctgtaatggccttaatgaaattcaagaccgttgatgaagctatccaaaaggcaaactgtactaagtacggtttagctgctggtatcgttactaaggaattgaatttggctaacacagtctccagaagtattagagccggtgctgtttgggtcaactgctattttggtttcgatccagacgctccttttggtggttacaaaatgtctggtttcggtaaagatagaggtgttttggcaatcgaccaatatttgaaggctaagacagttaacaccgcaattccaaactcaccttggtactaa |
| *ALD* from *Synechocystis* sp. PCC6803  (Syn_*ALD*) | atgaataccgccaaaactgttgtcgctgaacaaagagatttctttagacaaggtaaaactaagtctgttcaagacagattgaccgctttagcaaaattgaagactcaaatacaagctcaagaagaagaaattattaaggcattgaagcaagattttggtaaaccaactttcgaatcttacgtaaacgaaatcttgggtgttatcagagaaattaactactaccaaaaacatttgcaacaatggtccaagccacaaagagtcggtacaaatttgatggtatttcctgccagtgctcaattaagaccagaacctttaggtgtagttttgatcatttctccatggaactaccctttctacttgtgtttgatgccattgataggtgcaatcgctgctggtaactgcgtcgtagttaaaccatccgaatatactcctgccatcagtggtgttattacaagattgatccaaaacgtcttttccccagcatgggccacagtcgtagaaggtgacgaaaccatcagtcaacaattgttacaagaaaagttcgaccatatctttttcactggttcacctagagttggtagattgattatggctgctgctgctgaacaattaacaccagttaccttggaattaggtggtaaatccccttgtgttgtcgatagagaaatcaatttgcaagaaacagctaagagaataatgtggggtaaattggttaacgcaggtcaaacctgtgtcgccccagactatttgttggtagaacaatcatgcttggaacaattgttacctgctttgcaacaagcaatccaaatgttatttggtgaaaatccagctcactctcctgattacacaagaatcgttaaccaacaacaatggtcaagattggtatctttgttgtcacatggtaaagttataaccagaggtgaccataatgaaggtgacagatatatcgcaccaactttaataatcgatccagacttaaactcacctttgatgcaagaagaaatcttcggtccaatcttgcctatcttgacctaccaatccttgagtgaagccatagatttcatcaacatcaagccaaagcctttggctttgtactttttctctaacaacagacaaaagcaagaagaaatattgcaatctacatcttcaggttcagtttgcttgaacgatatcttgttgcatttgactgtcacagacttgccatttggtggtgttggtgaatctggtatgggtagatatcacggtaaagcaactttcgatacattgtccaactacaagagtatcttgagaagaccattctggggtgaaacaaatttgagatactctccttacggtaaaaagatgaacttaattaaaaagttgttttcataa |

**
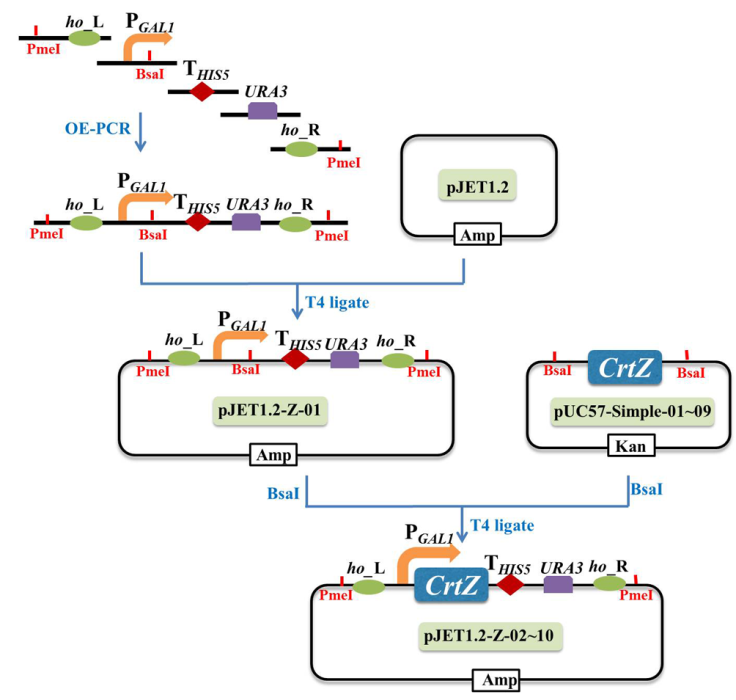
**

**Supplementary Figure 1.** Schematic representation of the engineering strategies for CrtZ expression cassette. *ho*_L represents *ho* locus left homologous arm, *ho*_R represents *ho* locus right homologous arm.

**
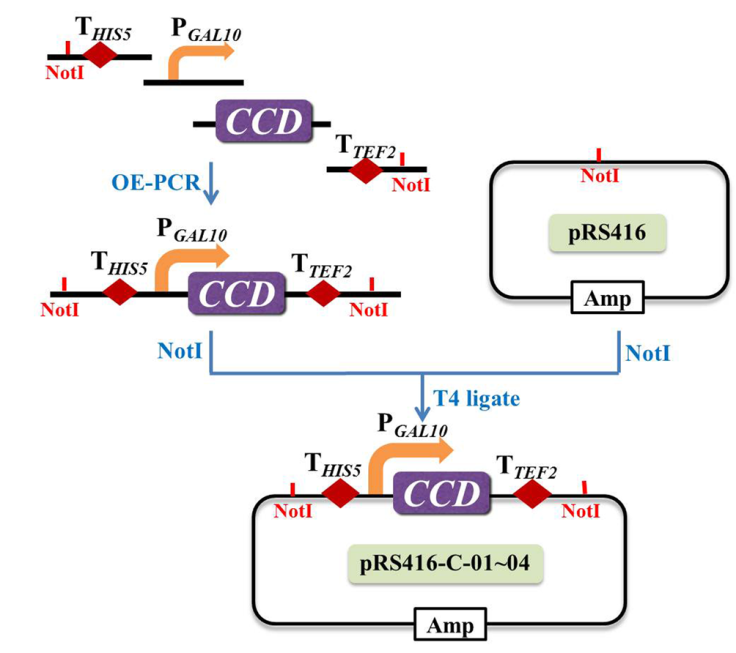
**

**Supplementary Figure 2.** Schematic representation of the engineering strategies for CCD expression cassette.

**
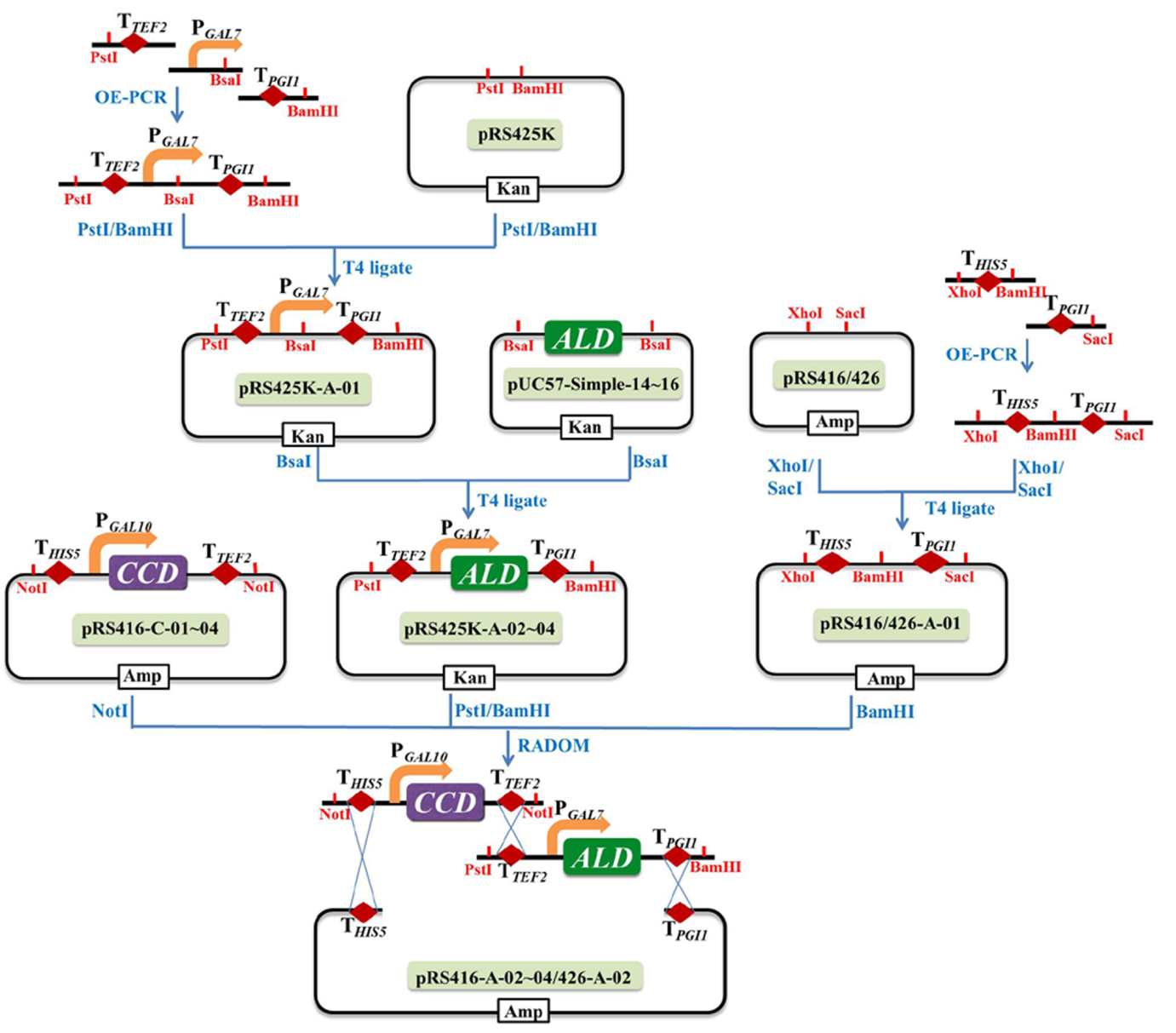
**

**Supplementary Figure 3.** Schematic representation of the engineering strategies for ALD expression cassette.


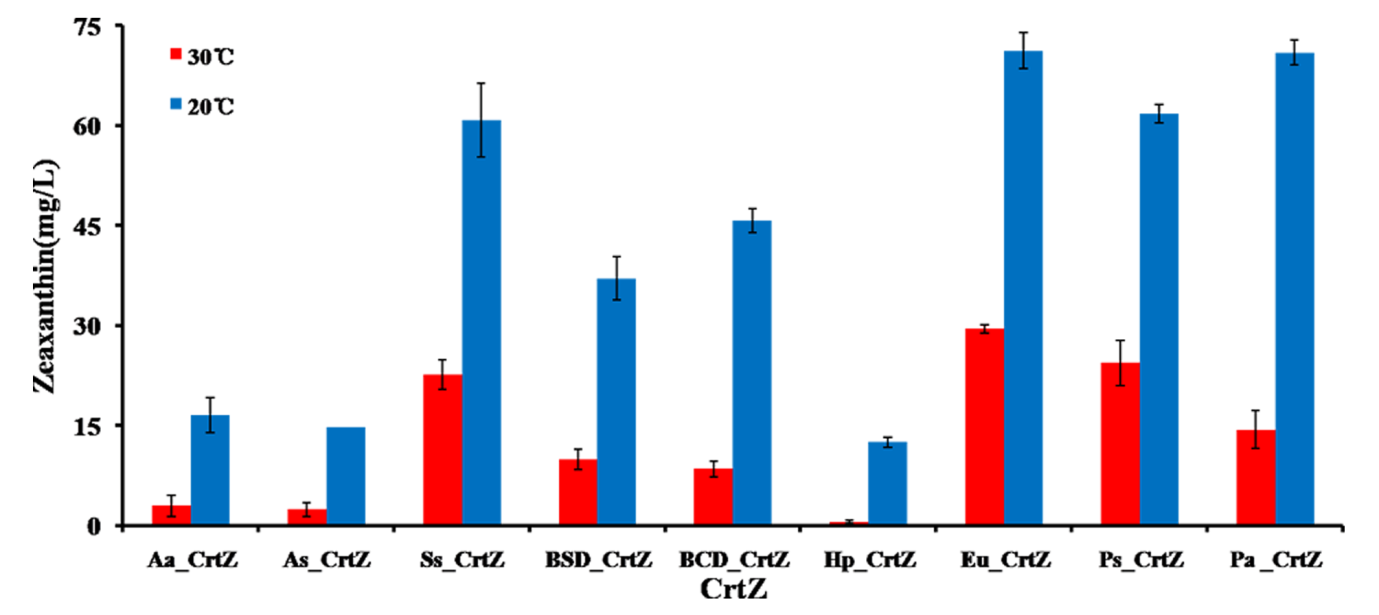


**Supplementary Figure 4.** The effect of temperature on zeaxanthin production in zeaxanthin producing strains. Production of zeaxanthin in shake-flask fermentation was analyzed by HPLC. The error bars represent standard deviation calculated from triplicate experiments. Abbreviations: Aa, *Agrobacterium aurantiacum*; As, *Alcaligenes* sp. PC-1. Eu, *E. uredovora*; Pa, *Pantoea agglomerans*; Ps, *Pantoea stewartii*; Ss, *Sulfolobus solfataricus* P2; B.SD, *Brevundimonas* sp. SD212; B.DC, *Brevundimonas vesicularis* DC263; Hp, *Haematococcus pluvialis*.


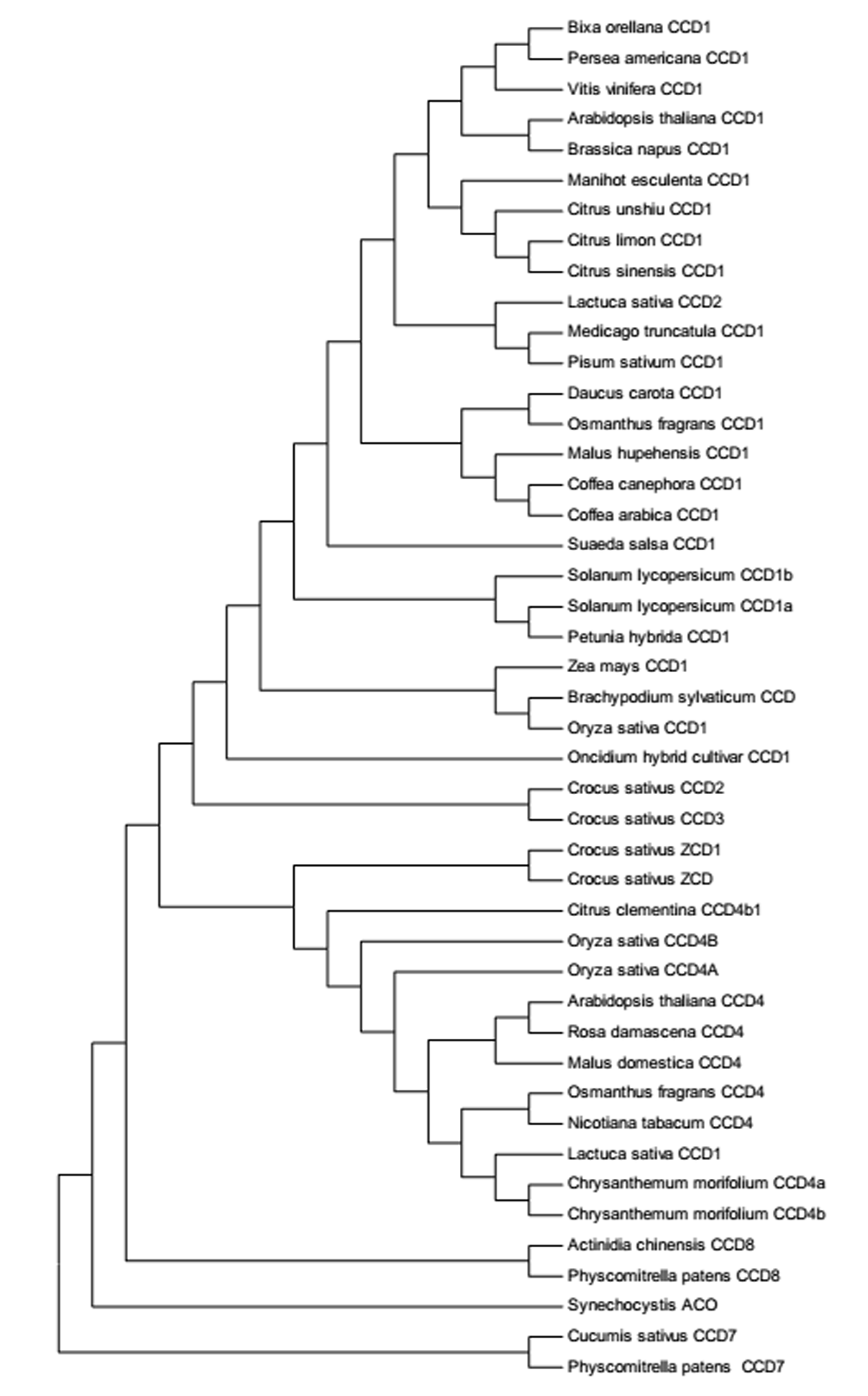


**Supplementary Figure 5.** Phylogenetic tree of CCD genes family was constructed and inferred by Neighbor-Joining method.


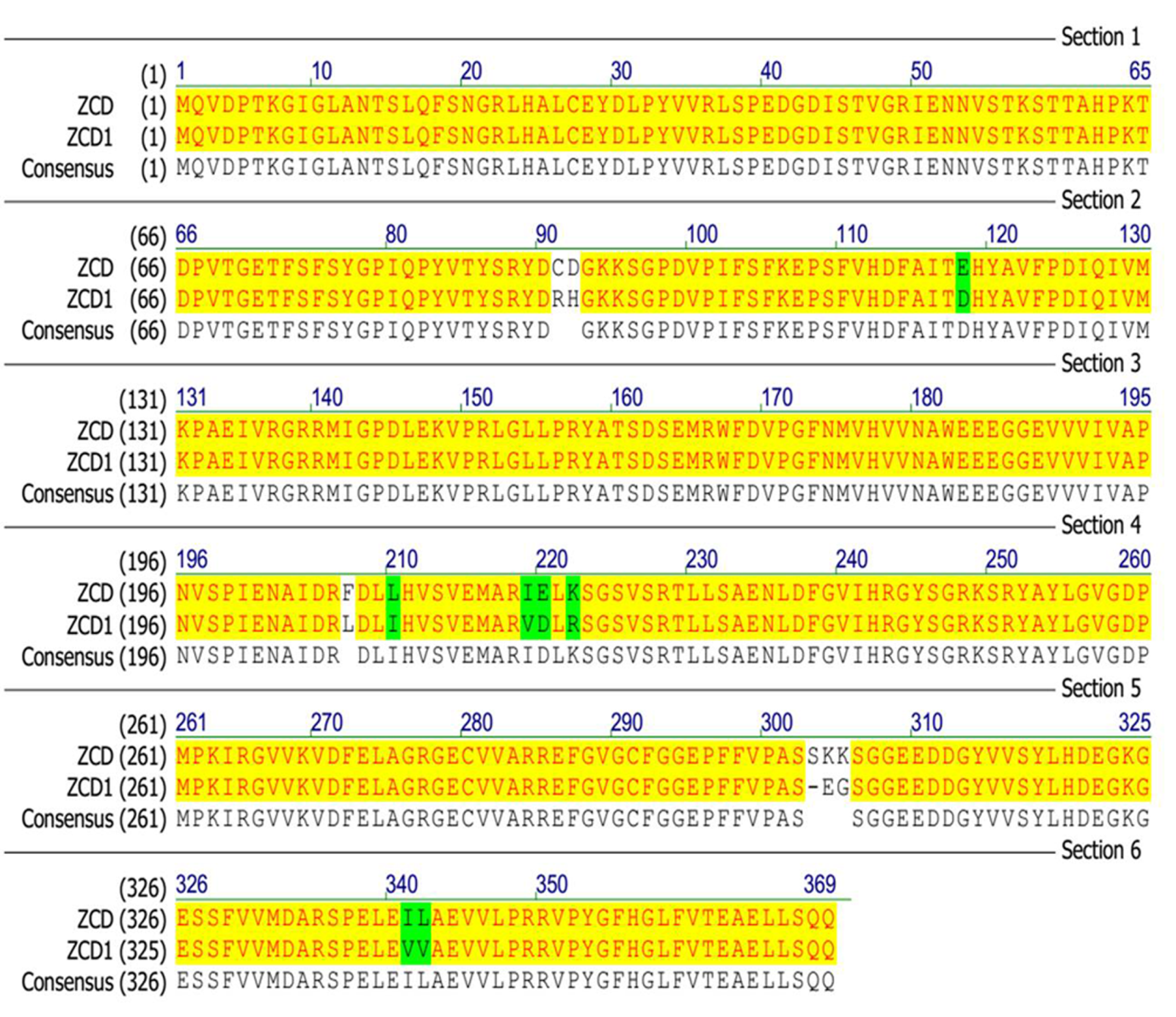


**Supplementary Figure 6.** Sequence alignment of ZCD and ZCD1 to identify the conserved region. Yellow: identical sequence. Green: block of similar.


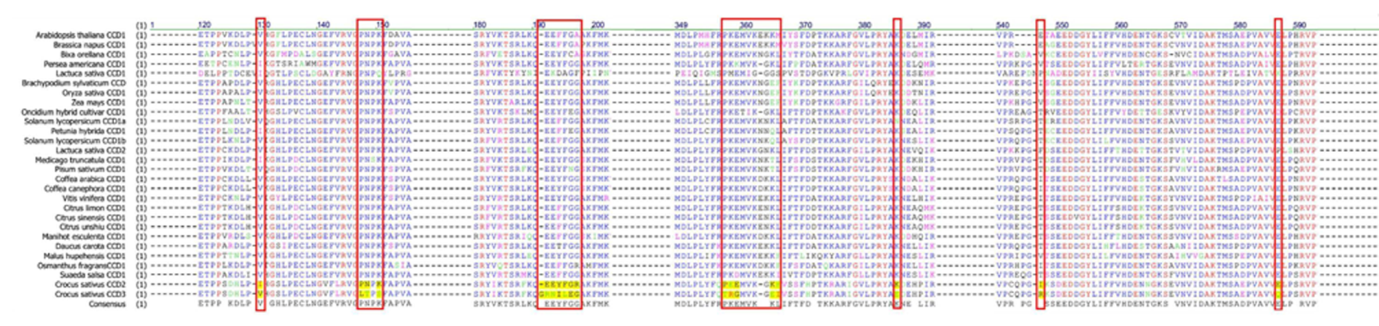


**Supplementary Figure 7.** Sequence alignment of CCD1 genes subfamily. Yellow: dissimilar fragments between CCD2 and CCD3. Red box: conservation analysis in dissimilar fragments between CCD2 and CCD3 based on sequence alignment of CCD1 genes subfamily.
